# Grapevine protein Src2 mediates plant disease resistance during *Lasiodiplodia theobromae* infection

**DOI:** 10.1093/plphys/kiaf608

**Published:** 2025-11-24

**Authors:** Junbo Peng, Qikai Xing, Wei Zhang, Xuncheng Wang, Hui Guo, Hui Wang, Chenfang Wang, Jiye Yan

**Affiliations:** Beijing Key Laboratory of Environment Friendly Management on Diseases and Pests of North China Fruits, Institute of Plant Protection, Beijing Academy of Agriculture and Forestry Sciences, Beijing 100097, China; Beijing Key Laboratory of Environment Friendly Management on Diseases and Pests of North China Fruits, Institute of Plant Protection, Beijing Academy of Agriculture and Forestry Sciences, Beijing 100097, China; Beijing Key Laboratory of Environment Friendly Management on Diseases and Pests of North China Fruits, Institute of Plant Protection, Beijing Academy of Agriculture and Forestry Sciences, Beijing 100097, China; Beijing Key Laboratory of Environment Friendly Management on Diseases and Pests of North China Fruits, Institute of Plant Protection, Beijing Academy of Agriculture and Forestry Sciences, Beijing 100097, China; Beijing Key Laboratory of Environment Friendly Management on Diseases and Pests of North China Fruits, Institute of Plant Protection, Beijing Academy of Agriculture and Forestry Sciences, Beijing 100097, China; Beijing Key Laboratory of Environment Friendly Management on Diseases and Pests of North China Fruits, Institute of Plant Protection, Beijing Academy of Agriculture and Forestry Sciences, Beijing 100097, China; Beijing Key Laboratory of Environment Friendly Management on Diseases and Pests of North China Fruits, Institute of Plant Protection, Beijing Academy of Agriculture and Forestry Sciences, Beijing 100097, China; Beijing Key Laboratory of Environment Friendly Management on Diseases and Pests of North China Fruits, Institute of Plant Protection, Beijing Academy of Agriculture and Forestry Sciences, Beijing 100097, China

## Abstract

Lysin motif (LysM) effectors contribute to the virulence of many pathogens, but the underlying mechanism of this type of effector remains poorly understood. Here, we identified a LysM-containing protein, named LtLysM2, in the plant opportunistic pathogen *Lasiodiplodia theobromae.* We demonstrated that LtLysM2 contributes to the virulence of the pathogen, is able to bind to chitin oligosaccharides, and can suppress chitin-triggered plant immune responses. Importantly, we showed that the grapevine (*Vitis vinifera*) protein VvSrc2, a membrane- and nuclear-localized protein homologous to the soybean (*Glycine max*) protein Src2 (Soybean genes regulated by cold 2), interacts with LtLysM2. Interestingly, the nuclear accumulation of VvSrc2 was elevated in the presence of LtLysM2. Ectopic expression of *VvSrc2* in *Nicotiana benthamiana* enhanced resistance to *L. theobromae*. Additionally, the VvSrc2 protein interacted with the nuclear-localized RNA-binding protein VvUbp1, contributing to the programmed cell death evoked by VvUbp1. Our findings reveal a previously uncharacterized regulatory pathway in which VvSrc2 is upregulated after the recognition of LtLysM2 and serves as an intermediator to transduce extracellular signaling events into nuclear components during infection by *L. theobromae* through association with the downstream target VvUbp1 in the cell nucleus.

## Teaser

Research on the regulatory mechanism between the effector secreted by the pathogen with the target proteins identified in grapevine contributes to the breeding of disease-resistant plants.

## Introduction

A diversity of plant pathogens, including bacteria, fungi, oomycetes, and nematodes, secrete an arsenal of effector proteins into apoplastic spaces or intracellular compartments to modulate host defense circuitry or enable parasitic colonization, thereby establishing successful infections (Hogenhout et al. 2009). Generally, effectors are described as secretory proteins and small molecules, including virulence or avirulence factors, toxins, and degradative enzymes, that alter the structure and function of host cells (Martin et al. 2003; Kamoun 2006; Hogenhout et al. 2009). Typically, effectors are regarded to be species or even lineage-specific; however, a growing number of recently identified effectors have been found to contain specific motifs (Kombrink et al. 2017). For instance, the RxLR and Crinkler (CRN) effectors in oomycetes were identified to possess an N-terminal RxLR domain and LxLFLAK motif, respectively (McGowan and Fitzpatrick 2017; Han and Kahmann 2019). Besides, the necrosis and ethylene-inducing protein (NEP1)-like proteins (NLPs), which generally induce cell death in dicotyledonous plants through plasma membrane permeabilization, are also widely distributed in many pathogenic bacteria, fungi, and oomycetes (Gijzen and Nürnberger 2006; Qutob et al. 2007; de Jonge et al. 2011; Dong et al. 2012). Moreover, the conserved fungal-specific extracellular membrane-spanning (CFEM) domain effectors are widely present in many fungi, including *Magnaporthe oryzae* (Kulkarni et al. 2003; Kou et al. 2017; Sabnam and Barman 2017), *Aspergillus fumigatus* (Vaknin et al. 2014), *Fusarium oxysporum* (Ling et al. 2015), and *Botrytis cinerea* (Zhu et al. 2017). Another group of effectors carrying the LysM domain is also widely distributed in a wide range of fungal species, including *Cladosporium fulvum* (de Jonge et al. 2010), *Mycosphaerella graminicola* (Marshall et al. 2011), *M. oryzae* (Mentlak et al. 2012), *Colletotrichum higginsianum* (Takahara et al. 2016), and *Verticillium dahliae* (Kombrink et al. 2017).

LysM was first discovered in the C terminus of the lysozyme of *Bacillus* phage Φ29 by Garvey et al. (1986). A typical LysM domain carries 44 to 65 amino acid residues, which are mainly located at the N- or C-termini and less frequently in the central region of LysM proteins (Buist et al. 2008; Visweswaran et al. 2014). To date, the functional mechanisms of LysM proteins have been widely documented in various species, including plants, fungi, and bacteria. In rice, the LysM protein OsCEBiP was confirmed to bind to chitin and subsequently led to the heterodimerization of OsCEBiP with its co-receptor OsCERK1, which resulted in the activation of plant immune responses (Shimizu et al. 2010; Tanaka et al. 2013). In the tomato pathogen *C. fulvum*, the LysM effector protein Ecp6 was found to contain three LysMs (LysM1, LysM2, and LysM3). Interestingly, the LysM1 and LysM3 can collectively bind to chitin with an ultrahigh (picomolar) chitin-binding affinity, which could sequestrate the fungal cell wall-derived chitooligosaccharides to protect these chitin fragments detected by plant immune receptors (de Jonge et al. 2010; Kombrink and Thomma 2013; Sánchez-Vallet et al. 2013; Akcapinar et al. 2015; Kombrink et al. 2017). Another characterized LysM effector, Slp1, in *M. oryzae* was reported to competitively bind to chitin with the rice pattern recognition receptor (PRR) protein CEBiP and suppress chitin-triggered immune responses (Mentlak et al. 2012). However, Slp1 could not provide protection from hyphal tip hydrolysis by chitinase enzymes, which was different from the characteristic of Avr4, another chitin-binding lectin found in *C. fulvum* (Kombrink and Thomma 2013; Rovenich et al. 2014; Akcapinar et al. 2015). Although the biological functions of LysM effectors are widely investigated in foliar and grain pathogens, their interactive targets from the host plant and the pathogenic mechanism of this type of effectors in the woody plant pathogens *Botryosphaeriaceae* family are barely documented.

Calcium ion serves as an important secondary messenger and responds to a diverse range of stimuli, including pathogen attack (Kim et al. 2008; Ma et al. 2009). Generally, activated Ca^2+^ signaling is coupled to downstream signal transduction pathways via Ca^2+^-binding proteins (Ma and Berkowitz 2011). The conserved C2 domain was originally identified as the second of 4 conserved domains (C1 to C4) in Ca^2+^-dependent isoforms of mammalian protein kinase C (PKC) and nowadays it is widely present in many Ca^2+^-binding proteins (Nalefski and Falke 1996; Kang et al. 2013). In animals, the domain was reported to be involved in multiple roles, including protein–protein interaction, binding of phospholipids, membrane and vesicular trafficking, and signal transduction (Davis et al. 1996; Cho and Stahelin 2006; Kang et al. 2013). For instance, the C2 domain of Rabphilin-3A identified in bovine acted as a modules for the interaction between this protein with its target and the interaction was dependent on phosphatidylserine and Ca^2+^ (Miyazaki et al. 1995). In canine. the C2 domain of ubiquitin protein ligase Nedd4 binds to phospholipids and membranes in a Ca^2+^-dependent fashion and is also involved in localizing the protein primarily to the apical region of polarized epithelial cells in response to Ca^2+^ (Plant et al. 1997). Other C2 domain proteins, such as Lgd identified in *Drosophila,* function as a critical regulator during endocytosis (Gallagher and Knoblich 2006). In comparison with the various reports of C2 domain-containing proteins identified in animals, the information related to C2 domain-containing proteins identified in plants remains relatively limited and a few publications have reported its role in plant response against biotic and abiotic stress. For example, in rice, a C2 domain-containing protein OsERG1 binds to phospholipid vesicles in a Ca^2+^-dependent manner and is translocated to the plasma membrane in response to either a fungal elicitor or a Ca^2+^ ionophore (Kim et al. 2003). In *Arabidopsis*, the C2 domain-containing proteins identified, such as BON1/CPN1 (Liu et al. 2005), BAP1, and BAP2 (Yang et al. 2006, 2007), play significant roles in repressing plant defense responses and general programmed cell death. Another nucleus-localized Ca^2+^-dependent lipid-binding protein (AtCLB) identified in *Arabidopsis* contains a C2 domain and negatively regulates plant responses to abiotic stress, including drought and salt tolerance (de Silva et al. 2011). In mung bean, the C2 domain-containing protein Vr-PLC3 is upregulated by drought or salt stress stimulation in an ABA-independent manner, and importantly, the C-terminal C2 domain is critical for its membrane localization (Kim et al. 2004). Another C2 domain-containing protein CaSRC2-1, a homolog to the Soybean protein Src2 (Soybean genes regulated by *c*old), responds to pathogen attack as well as abiotic stressor (Takahashi and Shimosaka 1997; Kim et al. 2008). Function characterization revealed that CaSRC2-1 was required for *Phytophthora capsici* INF1-triggered immunity by acting as an interacting partner of PcINF1 and, similarly, the C2 domain is crucial for the membrane localization of CaSRC2-1 (Kim et al. 2008; Liu et al. 2015, 2016). Recently, a Src2 protein identified in *Nicotiana benthamiana* was reported to play a positive role in resistance to *Cucumber mosaic virus* (CMV) infection (Saikia et al. 2025). In terms of their crucial roles in plant immunity, our understanding of the regulatory mechanism behind its molecular function remains unrevealed to a large extent.

*Botryosphaeriaceous* fungi are cosmopolitan in distribution and occur on a wide range of plant species, such as monocotyledonous, dicotyledonous, and gymnosperm hosts, and on multiple plant organs, including woody branches, herbaceous leaves, stems and culms of grasses, twigs, and lichen thalli (Liu et al. 2012). So far, among the *Botryosphaeriaceae* family members, over 20 have been found to be capable of causing grapevine dieback and cankers globally (Crous et al. 2006; Úrbez-Torres 2011; Úrbez-Torres et al. 2012; Phillips et al. 2013; Yan et al. 2013). Although we have got relatively better understanding of the infection characteristics and lifestyles, the pathogenic mechanisms of *Botryosphaeriaceae* fungi remain ambiguous. In a previous study, we performed a systematic secretome analysis of *L. theobromae*, recorded as a very virulent strain of *Botryosphaeriaceae* family in China. Bioinformatic analyses revealed that *L. theobromae* strain carried over 900 presumably secretory proteins, including 357 putative effector proteins involved in various pathogenicity-related pathways (Yan et al. 2018); however, the concrete functions of these effector proteins are yet to be investigated in depth.

In this study, we characterized a LysM effector LtLysM2 in *L. theobromae* in detail. It was found that LtLysM2 contributed to the virulence of the pathogen, and suppressed chitin-triggered defense response. Importantly, we identified a membrane and nuclear-localized protein, VvSrc2, that interacted with LtLysM2 and interfered with the immunity-inhibitive function of LtLysM2. Additionally, overexpression of *LtLysM2* promoted the nuclear accumulation of VvSrc2, and transient expression of *VvSrc2*, conversely, enhanced the resistance of *N. benthamiana to L. theobromae*. Interestingly, the VvSrc2 protein also interacted with an RRM domain-containing protein VvUbp1 in the nucleus and enhanced the intensity of programmed cell death evoked by VvUbp1. Our findings uncovered a regulatory pathway in which the VvSrc2 protein was upregulated after the perception of LtLysM2 and also inhibited the immunity-suppressive function of LtLysM2; on the other hand, VvSrc2 may serve as a signal transducer to transduce the upstream signal events into nuclear components to mount plant defense response by the association with downstream target VvUbp1 in the nucleus.

## Results

### Systematic identification of LysM effector homologs in the proteome of *L. theobromae*

In our previous study, over 900 secreted proteins were identified in the whole proteome of *L. theobromae* (Yan et al. 2018). Among them, 6 proteins containing various LysM domains were identified by BLASTp using the amino acid sequence of *C. fulvum* Ecp6 as the query subject. Phylogenetic analyses of 6 LysM proteins with other reported LysM proteins, including Slp1 and Slp2 in *Magnaprothe oryzae*, Ecp6 in *C. fulvum*, ChELP1 and ChELP2 in *C. higginsianum*, and Mg1LysM, Mg3LysM, and MgxLysM in *M. graminicola*, revealed their relatedness to each other (Supplementary Fig. S1A). In addition, the phylogram showed that LtLysM1 and LtLysM3 belong to group I, along with ChELP1, ChELP2, CfEcp6, MgSlp1, and Mg3LysM, and LtLysM2, LtLysM4, LtLysM5, and LtLysM6 are members of group II. It is worth noting that LtLysM1, LtLysM2, and LtLysM3 were predicted to be effector proteins (Supplementary Table S1). As the LtLysM1 and LtLysM3 (referred to as LtScp1 in the publication) have been documented in previous reports, we attend to a functional characterization for LtLysM2 in the study.

### LtLysM2 contributes to the virulence of *L. theobromae*

**Figure 1.**
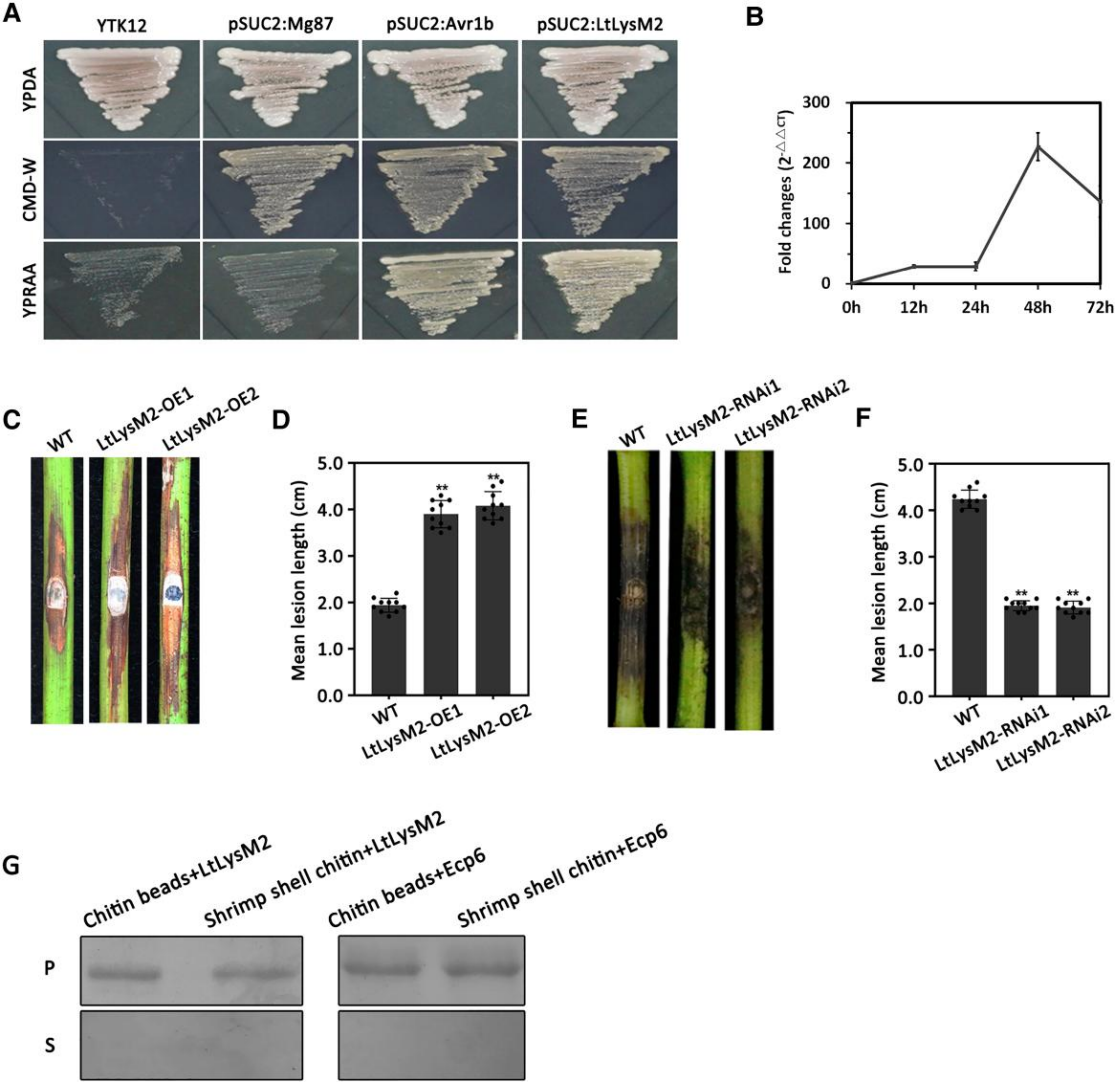
LtLysM2 contributed to the virulence of *L. theobromae*. **A)** Functional validation of LtLysM2 signal peptide (SP) using a yeast invertase secretion assay. The predicted LtLysM2 SP was capable of guiding the secretion of yeast invertase, and therefore, yeast transformants expressing *pSUC2:LtLysM2SP* (in which LtLysM2 SP was in frame to yeast invertase) could grow on YPRAA media with raffinose as the sole carbon source and CMD-W media. The N-terminal amino acid sequences of Avr1b from *P. sojae* and of Mg87 from *M. oryzae* were used as positive and negative controls, respectively. Additionally, transformed yeast cells were able to grow on CMD-W media. The parental strain YTK12 used in this study was not able to grow on YPRAA and CMD-W media. **B)** Relative transcript levels of *LtLysM2* at different infectious stages. The *L. theobromae-*infected grapevines were harvested at 12, 24, 48, and 72 h post-inoculation (hpi) for *LtLysM2* gene expression analyses using qRT-PCR assays. The *actin* gene was used as the internal control. Relative transcript levels of *LtLysM2* at infectious states were normalized by the *actin* gene and calibrated against those of mycelia. Relative transcript levels were calculated using the 2^−ΔΔCT^ method. Two biological repetitions with 3 replicates were assayed, and a representative set of data is presented. Data are means ± Se. **C–F)** Pathogenicity test of the overexpressed transformants (LtLysM2-OE1, LtLysM2-OE2) and silenced transformants of LtLysM2 (LtLysM2-RNAi1, LtLysM2-RNAi2), as well as wild type (WT). The wounded grapevine shoots were inoculated with mycelial plugs (5 mm in diameter) of overexpressed transformants **(C)** and silenced transformants **(E)**, and then kept in a chamber under constant humidity and temperature. The inoculated grapevines were photographed at 3 d post-inoculation. Statistical analyses of lesion lengths caused by the overexpressed transformants **(D)** and silenced transformants **(F)**. Three repetitions were performed independently and 5 replicates were performed each. A representative set of data is presented. Error bars represent Sd of 3 replicates. Significant differences were evaluated using the one-way analysis of variance (ANOVA) and least significant difference (LSD) tests (**α = 0.01). **G)** Affinity precipitation of LtLysM2 with chitin beads and chitin from shrimp shells. Following centrifugation, the pellet fractions (P) and supernatant fractions (S) were collected and analyzed by SDS–PAGE and Coomassie brilliant blue staining. Detection of LtLysM2 in the pellet fraction indicates that LtLysM2 is capable of binding to chitin polysaccharide. The Ecp6 protein from *C. fulvum* was used as the control.

According to the amino SignalP 5.0 program, LtLysM2 was predicted to possess a signal peptide (SP) with 17 amino acids (Supplementary Fig. S1B). To confirm the function of LtLysM2 SP, an elegant system based on the requirement of invertase secretion for yeast cells to grow on media with raffinose as the sole carbon source was adopted (Jacobs et al. 1997; Fang et al. 2016). In the assay, we engineered a *pSUC2:LtLysM2SP* fusion construct in which the yeast invertase lacking its own SP was introduced one amino acid after the predicted LtLysM2 SP. Fusion vectors carrying the N-terminal sequences of *Phytophthora sojae* Avr1b and *M. oryzae* Mg87 were used as positive and negative controls, respectively. Subsequently, the fusion vector was transformed into the invertase secretion-deficient yeast strain YTK12. Same to positive control, yeast transformants carrying *pSUC2:LtLysM2SP* grew on YPDA, CMD-W, and YPRAA media; negative control grew on YPDA and CMD-W media but not on YPRAA media (Fig. 1A).

As LtLysM2 was confirmed to be a secreted protein, we set out to examine its role in the virulence of *L. theobromae*. Initially, we quantified the relative transcript levels of the *LtLysM2* gene during the infection by RT-qPCR. Statistical data showed that the transcript level of *LtLysM2* increased considerably at 48 hpi, followed by a decrease at 72 hpi (Fig. 1B). Following the transcription profiling, we performed the targeted gene overexpression and silencing of *LtLysM2* to decipher its virulent roles during disease development. Two transformants of each type (hereinafter referred to as LtLysM2-OE1, LtLysM2-OE2, LtLysM2-RNAi1, and LtLysM2-RNAi2) confirmed via qRT-PCR and neomycin resistance screening were selected for the subsequent pathogenicity test. Mycelial plugs of LtLysM2-OE (LtLysM2-OE1 and LtLysM2-OE2) and LtLysM2-RNAi transformants (LtLysM2-RNAi1 and LtLysM2-RNAi2), as well as the isogenic wild type strain CSS-01s were collected and applied to 1-yr-old green shoots of susceptible grapevine cultivar “Summer Black,”. The obtained results showed that lesion length of grapevines inoculated with LtLysM2-OE transformants were obviously increased relative to that of wild-type strain CSS-01s, which was further quantified by statistically analyses (Fig. 1C and D); by contrast, and the LtLysM2-RNAi transformants caused attenuated disease symptoms in the grapevine shoots (Fig. 1E and F), implying that *LtLysM2* was conducive to the virulence of *L. theobromae* during the symptom development.

One of the key features of LysM effector proteins is their ability to bind to chitin oligosaccharides. As LtLysM2 was predicted to contain 2 LysM domains (Supplementary Fig. S1B), we decided to examine whether this protein was capable of binding to chitin by affinity precipitation assays. The *Escherichia coli*-generated LtLysM2 protein was incubated with the insoluble polysaccharides shrimp shell chitin and chitin beads. It was found that, similar to the LysM domain-containing protein Ecp6 identified in *C. fulvum* (de Jonge et al. 2010), LtLysM2 was precipitated with the 2 carbohydrates tested and detected in the insoluble polysaccharide pellet after SDS–PAGE and Coomassie brilliant blue staining (Fig. 1G), suggesting that LtLysM2 is a chitin-binding protein.

### LtLysM2 associated with VvChi4 and VvSrc2

**Figure 2.**
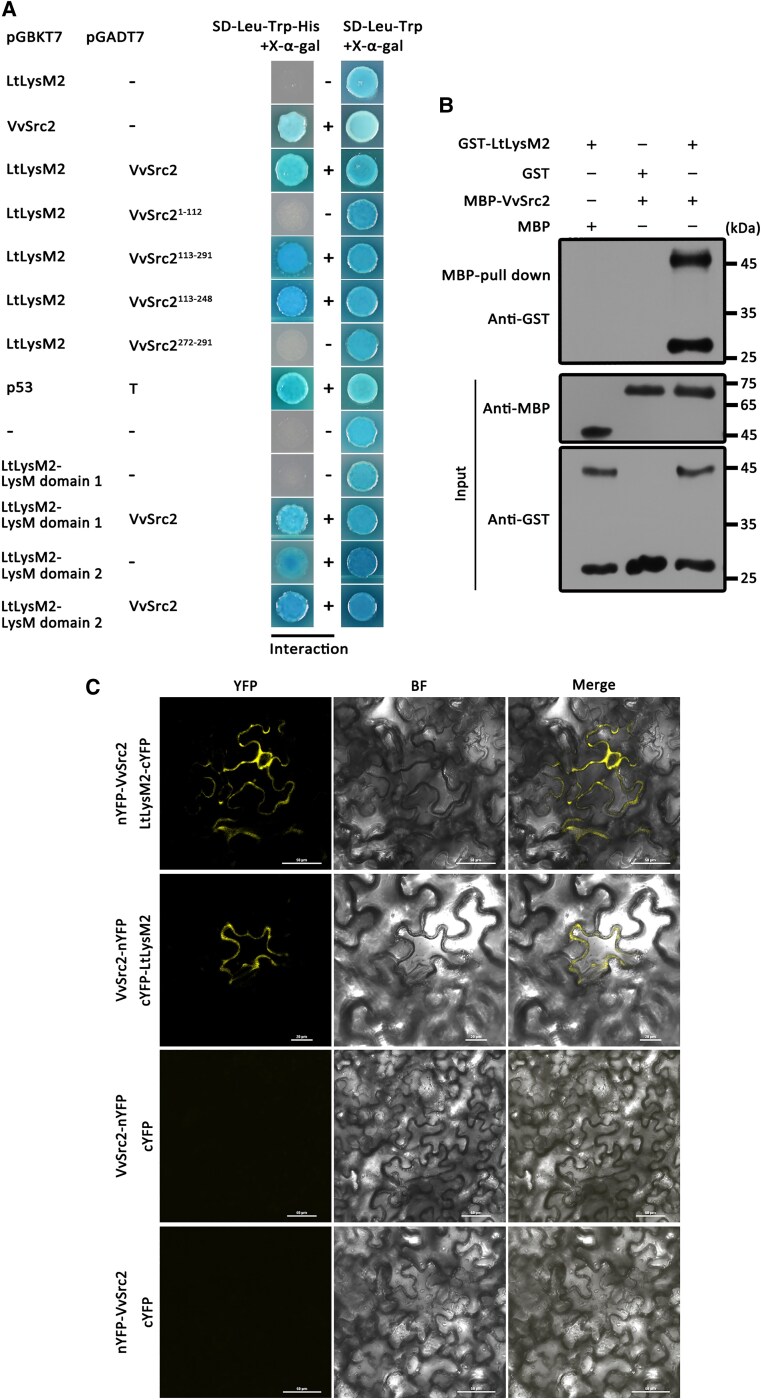
LtLysM2 is associated with VvSrc2. **A)** Yeast two-hybrid illustrating the LtLysM2 molecule interacting with VvSrc2. Yeast cell expressing each bait/prey pair was assayed for growth on auxotrophic media lacking leucine(-Leu), tryptophan(-Trp), and histidine(-His) and for X-*α*-galactosidase activities. Yeast cells expressing the empty vector *pGADT7* with empty vector *pGBKT7*, or with the bait construct of *LtLysM2,* were used as negative controls. The interaction between *pGADT7-T* with *pGBKT7-53* was used as the positive control. The symbols +and − marked the presence of interaction and absence of interaction, respectively. LtLysM2-LysM domain 1 marks the first LysM domain of the LtLysM2 protein. LtLysM2-LysM domain 2 marks the second LysM domain of the LtLysM2 protein. SD, synthetic dropout. **B)** Interaction validation of VvSrc2 with LtLysM2 using MBP pull-down experiments. The recombinant proteins GST-LtLysM2 and MBP-VvSrc2 purified from *E. coli* were subjected to MBP pull-down analyses. The interacting proteins were detected with an immunoblot. **C)** In planta confirmation of LtLysM2/VvSrc2 interaction using a bimolecular fluorescence complementation (BiFC) assay in *N. benthamiana*. The LtLysM2 and VvSrc2 were fused to the N-terminus and C-terminus of YFP epitope tag, respectively. Fluorescence signal was detected with a confocal microscope 2 d post-infiltration (dpi). BF, bright field.

To identify the potential targets with which LtLysM2 could interact, we performed a yeast two-hybrid screening against grapevine cDNA library with LtLysM2^ΔSP^ as the bait. A total of 6 candidates that potentially interacted with LtLysM2 were obtained (Supplementary Table S2). As the LysM domain-containing effector was supposed to be an apoplastic protein, and thereby, based on subcellular localization prediction, a putatively secreted class IV chitinase VvChi4 and an Src2-like protein VvSrc2, putatively located to the cell membrane and nucleus, were selected for subsequent analyses. As expected, both proteins were confirmed to interact with LtLysM2 using the yeast two-hybrid system (Fig. 2A; Supplementary Fig. S2A).

To map the key region of VvChi4 and VvSrc2 that interacted with LtLysM2, a series of truncated forms of VvChi4 and VvSrc2 were generated dependent on their predicted domains and tested for their interaction with the effector by yeast two-hybrid. The obtained results demonstrated that only yeast transformants expressing the Glyco_hydro_19 domain (amino acid sequence from 55 to 216) of VvChi4 grew on the selective medium and displayed the ability to hydrolyze X-α-gal, suggesting the domain was the essential region that interacted with LtLysM2 (Supplementary Fig. S2A). In terms of VvSrc2, yeast cells expressing the Src2^1-112^ (C2 domain) did not grow on the selective medium and the fusion constructs expressing the fragment or Src2^113-291^ (deletion of the C2 domain) enabled the growth of yeast on the selective medium and the development of blue coloration when treated with the X-α-gal agent. Further investigation revealed that, for the moiety of Src2^113-291^, a shorter fragment Src2^113-248^ (deletion of the C2 domain, the transmembrane domain and the C-terminal end), interacted with LtLysM2 protein. Interestingly, it was found that both LysM domains of the LtLysM2 protein interacted with VvSrc2 (Fig. 2A).

Furthermore, to supply more convincing evidence to validate the interaction between LtLysM2 with VvChi4 or with VvSrc2, MBP pull-down assays were performed. The recombinant GST-LtLysM2 protein was purified from *E. coli* with glutathione agarose and incubated with MBP-VvChi4 or with MBP-VvSrc2 proteins purified with MBP magnetic beads. Protein immunoblotting analyses showed that GST-LtLysM2 was pulled down by MBP-VvChi4 and MBP-VvSrc2 protein, respectively (Fig. 2B; Supplementary Fig. S2B). Additionally, we adopted a bimolecular fluorescence complementation (BiFC) approach to examine the molecular interaction of LtLysM2 and VvSrc2 in vivo. An N-terminal fragment of YFP was fused to LtLysM2 and co-expressed with a C-terminal fragment of YFP fused to VvSrc2. Yellow fluorescence, indicative of YFP recombination, was observed between LtLysM2 and VvSrc2 (Fig. 2C). The results strongly support that VvChi4 and VvSrc2 are 2 targets with which LtLysM2 interacts. As the interaction between VvChi4 and another LysM effector has been documented previously, we select the VvSrc2 protein for further exploration subsequently.

### LtLysM2 promoted the protein accumulation of VvSrc2 in plant

**Figure 3.**
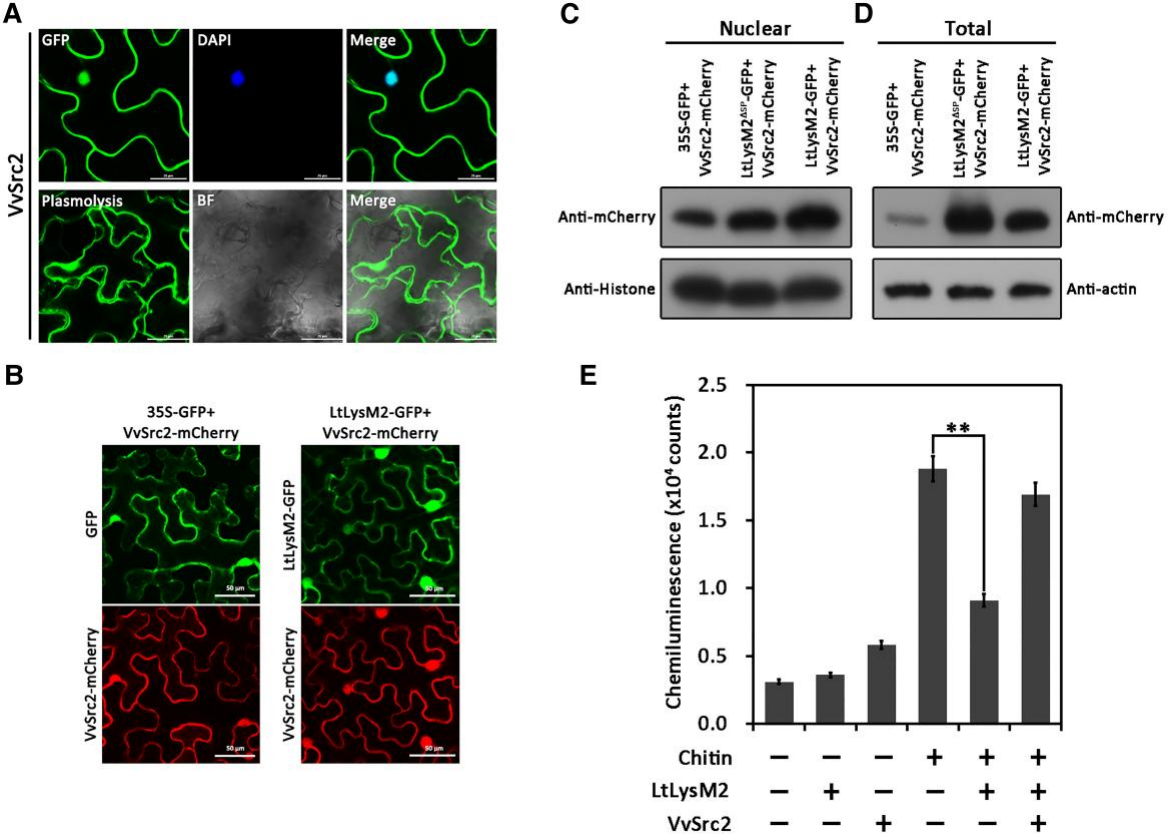
LtLysM2 promoted the expression of VvSrc2 in plants. **A)** Subcellular localization detection of VvSrc2 in plants. *Agrobacterium tumefaciens*-mediated transient expression of VvSrc2-GFP uncovered that VvSrc2 localized to the cell membrane and nucleus, which was substantiated by salt-induced plasmolysis and DAPI staining, respectively. BF, bright field. **B)** Subcellular localization detection of VvSrc2 in the presence/absence of LtLysM2. The intensity of red fluorescence from *N. benthamiana* nucleus co-expressing LtLysM2 and VvSrc2 was stronger relative to that of *N. benthamiana* co-expressing the empty vector and VvSrc2. Fluorescence signals were detected on a Zeiss 710 confocal microscope at an excitation wavelength of 488 nm (for green fluorescence detection) or 561 nm (for red fluorescence detection). **C, D)** Immunoblot analyses of the nuclear protein (**(C)** and total protein **(D)** isolated from the *N. benthamiana* samples mentioned in **(B)**. **E)** VvSrc2 inhibited the immunity-suppressive function of LtLysM2. Purified LtLysM2 protein (1.2 mg/mL) or VvSrc2 protein (1.2 mg/mL) was incubated with 0.2 mg/mL chitin in the solution composed of 50 mm Tris–HCl (pH 7.0) and 100 mm NaCl for 10 min with constant shaking at 4 °C. Subsequently, the reaction was supplied with the ROS reaction mixture to detect the luminescence. Error bars represent SD of 3 replicates. Significant differences were evaluated using the one-way analysis of variance (ANOVA) and least significant difference (LSD) tests (**α = 0.01).

Based on the Pfam program analyses, VvSrc2 was predicted to contain a C2 domain, a transmembrane (TM) domain, and a nuclear localization signal (NLS) motif (Supplementary Fig. S3). To understand the molecular function of VvSrc2, we initially investigated the subcellular localization of VvSrc2 by transiently expressing GFP-VvSrc2 in *N. benthamiana*. As expected, the green fluorescence of GFP-VvSrc2 was accumulated in cell membrane and nucleus, which were substantiated by a salt-induced plasmolysis of the epidermal cell and DAPI staining, respectively (Fig. 3A). To investigate whether the specific domain of VvSrc2 has an effect on its subcellular localization, we engineered a series of truncated fragments that were transiently expressed in *N. benthamiana* to detect the fluorescence signals. Similar to VvSrc2-GFP, the green fluorescence of VvSrc2-ΔTM-GFP mutant was distributed in the nucleus and membrane. The VvSrc2-nls-GFP mutant, however, was localized to the cell membrane; the fluorescence signals from VvSrc2-ΔC2-GFP and VvSrc2-ΔC2/ΔTM-GFP mutants were mainly distributed in the nucleus and also gathered into small clumps (Supplementary Fig. S4A). These results evidenced that the NLS motif and C2 domain of VvSrc2 were associated with its nuclear and membrane localization, respectively. The TM domain, however, does not affect the subcellular localization of VvSrc2. In each case, an immunoblot of the construct used for subcellular localization detection was performed to confirm the expression of each protein (Supplementary Fig. S4B).

The interactive association between LtLysM2 and VvSrc2 prompted us to address the way by which LtLysM2 affected VvSrc2 in plants. As VvSrc2 is confirmed to be a membrane and nucleus localized protein, we first sought to investigate whether the subcellular accumulation of VvSrc2 in plants was affected by LtLysM2. Notably, it was observed that the intensity of nuclear-localized red fluorescence emitted by VvSrc2-mCherry protein was relatively stronger in *N. benthamiana* leaves transiently expressed VvSrc2-mCherry and LtLysM2-GFP simultaneously, in comparison with that of control, suggesting that the expression of LtLysM2 contributed to the nuclear accumulation of VvSrc2-mCherry in plants (Fig. 3B). To verify the above observation, we performed immunoblot analyses with an anti-mCherry antibody to examine the nuclear amount of VvSrc2-mCherry in the presence/absence of LtLysM2. In consistent with the results evidenced by microscopy, the nuclear amount of VvSrc2-mCherry protein isolated from *N. benthamiana* leaves coexpressing VvSrc2-mCherry and LtLysM2-GFP protein was visibly elevated compared to that of the control (Fig. 3C). Additionally, the total amount of VvSrc2-mCherry protein was also increased in *N. benthamiana* leaves co-expressing both proteins simultaneously (Fig. 3D), suggesting that the protein amount of VvSrc2 were upregulated in the presence of LtLysM2.

Because LtLysM2 binds to chitin, we therefore tested to examine whether LtLysM2 suppressed chitin-triggered ROS production as other LysM proteins does (de Jonge et al. 2010; Mentlak et al. 2012). Similarly, LtLysM2 suppressed chitin-activated reactive oxygen species (ROS) burst in *N. benthamiana* cells, as shown in Fig. 3E. Interestingly, the suppression of chitin-triggered ROS by LtLysM2 was inhibited by its interactor VvSrc2, implying that VvSrc2 may compete with chitin to bind to LtLysM2 through occupancy effects, to attenuate the immunity-inhibitive function of LtLysM2 for disease progression.

### Overexpression of *VvSrc2* enhanced plant immunity

**Figure 4.**
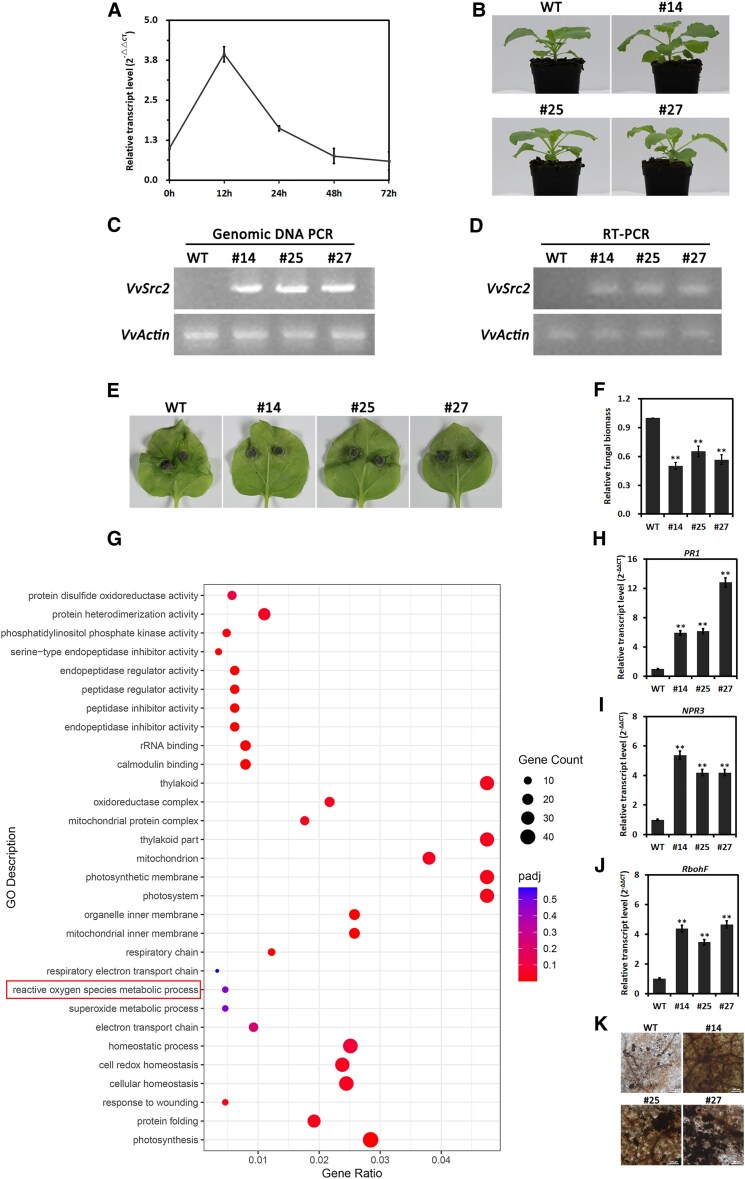
VvSrc2 enhanced the resistance of *N. benthamiana* to *L. theobromae*. **A)** Expression profiles of *VvSrc2* during the infection processes. The *L. theobromae-*inoculated grapevines were harvested at 12, 24, 48, and 72 h post-inoculation (hpi) for *VvSrc2* expression analyses using qRT-PCR assays. The *actin* gene was used as the internal control. Relative transcript levels of *VvSrc2* genes at different infectious states were normalized by the *actin* gene and calibrated against those of the uninoculated grapevines. Relative transcript levels were calculated using the 2^−ΔΔCT^ method. Two biological repetitions with 3 replicates were assayed, and a representative set of data is presented. Data are means ± Se. **B)** The growth phenotype of wild type (WT) and *VvSrc2*-transgenic lines (#14, #15, #27) at 4 wk post-germination. **C**, **D)** Confirmation of the transgene integration by genomic PCR and RT-PCR. **E)** Typical leaf symptoms of wild type (WT) and *VvSrc2*-transgenic lines (#14, #15, #27) infected by *L. theobromae.*  **F)** Relative fungal biomass of *L. theobromae* determined by qPCR at 48 h post-inoculation (hpi). Data are means ± Se of 3 replicates. Significant differences were evaluated using the one-way analysis of variance (ANOVA) and least significant difference (LSD) tests (**α = 0.01). **G)** Gene ontology (GO) classification of enriched DEGs identified from pairwise comparison between wild type and *VvSrc2*-transgenic line infected by *L. theobromae*. **H-J)** Transcription examination of resistance-related genes by RT-qPCR. The experiment and data analyses were performed using similar methods mentioned in **(A)**. Significant differences were evaluated using the one-way analysis of variance (ANOVA) and least significant difference (LSD) tests (**α = 0.01). **K)** ROS detection of *L. theobromae-*infected *N. benthamiana* leaves by staining with a DAB agent 2 d post-inoculation (dpi).

After confirming the interaction between LtLysM2 with VvSrc2, we sought to investigate whether the product of VvSrc2 had a role in regulating plant immunity during infection. First, we investigated the expression profiles of *VvSrc2* during the infection processes using RT-qPCR. Statistical data showed that the relative transcript levels of *VvSrc2* were significantly elevated during infection and reached a peak at the early-infectious stage (Fig. 4A), implicating that *VvSrc2* may partake in the response to pathogen infection and be involved in host defense response.

Following that, we engineered a VvSrc2 expressing construct driven by the cauliflower mosaic virus (CaMV) 35S promoter, and transformed the fusion vector into *N. benthamiana* via *Agrobacterium*-mediated leaf disc transformation. A total of 10 independent transgenic lines were obtained based on kanamycin resistance selection. As for *VvSrc2*-transgenic lines, 3 representative lines (#14, #15, and #27) with high expression levels were selected for subsequent exploration. Phenotype observations showed that, in terms of plant morphology, no obvious alterations were detected between the *VvSrc2*-trangenic line and wild type (Fig. 4B). Transgene integration and expression confirmation were performed for the T3 transgenic lines by genomic DNA PCR and RT-PCR assays (Fig. 4C and D). Subsequently, leaves detached from transgenic lines were assayed for disease development infected by *L. theobromae*. Therefore, we inoculated *N. benthamiana* leaves with mycelial plugs of *L. theobromae* and assessed the diseased lesion 3 d post-inoculation (dpi). All the inoculated leaves displayed water-soaked symptoms, and the expression of *VvSrc2-*transgenic lines displayed attenuated disease symptoms compared to the wild-type, indicating that VvSrc2 suppressed the disease development caused by the pathogen (Fig. 4E). To obtain more precise detection of disease-inhibitive function of VvSrc2 during infection, we measured the ratio of *L. theobromae* DNA to *N. benthamiana* DNA by qPCR to quantify the fungal biomass. The *L. theobromae* biomass of *N. benthamiana* expressing *VvSrc2* was relatively lower than that of the wild type (Fig. 4F), demonstrating that VvSrc2 suppressed the disease development caused by *L. theobromae.*

The attenuated symptoms of the transgenic lines prompted us to address whether the differentiation was associated with host immune responses. For this, we profiled the transcriptomes of the wild type and *VvSrc2*-transgenic line based on the Illumina next-generation sequencing technology. Here, the wild type and transgenic line either grew under normal conditions or were inoculated with *L. theobromae* mycelial plugs, and thereby, a total of 4 group samples were generated for RNA-seq. Reads were aligned to the reference genome of *N. benthamiana*. Pairwise transcriptome comparisons were performed between the wild type and the *VvSrc2*-transgenic line. As for the wild type and VvSrc2-transgenic line pair without inoculation (WT_N vs VvSrc2_N), a total of 4,693 differentially expressed genes (DEGs, 2016 genes upregulated and 2,677 genes downregulated) were identified (Supplementary Fig. S5). In term of wild type and VvSrc2-transgenic line infected by *L. theobromae* (WT_Y vs VvSrc2_Y), a total of 5556 DEGs (2,630 genes upregulated and 2,926 genes downregulated) were identified (Supplementary Fig. S6). Gene ontology (GO) annotation on WT_Y vs VvSrc2_Y revealed that, among the DEGs of WT_Y vs VvSrc2_Y, biological processes involved in photosynthesis, cellular homeostasis, and superoxide metabolic process etc. were represented. In terms of cellular component category, the respiratory chain, photosystem, mitochondrion, thylakoid, and oxidoreductase complex were significantly enriched. With regard to molecular function, calmodulin binding, rRNA binding, and phosphatidylinositol phosphate kinase activity etc. were significantly represented (Fig. 4G; Supplementary Table S3). To verify the reliability of the RNA-seq transcriptome data, the relative transcript abundances of 3 genes related to SA biogenesis and ROS anabolism were tested by RT-qPCR with specific primers. The obtained data showed that the transcript accumulation of these genes was significantly elevated in the transgenic line in comparison with that of the wild type (Fig. 4H to J). Additionally, we detected the accumulated level of ROS of the wild type and the *VvSrc2*-transgenic line infected by the pathogen. Microscopy observation revealed that, compared to wild type, a higher level of ROS accumulated in the transgenic lines as detected by 3,3-diaminobenzidine (DAB) staining (Fig. 4K). Collectively, it is reasoned that VvSrc2 functions as a positive regulator in mediating plant defense response during plant-pathogen interaction.

### VvSrc2 interacts with VvUbp1

**Figure 5.**
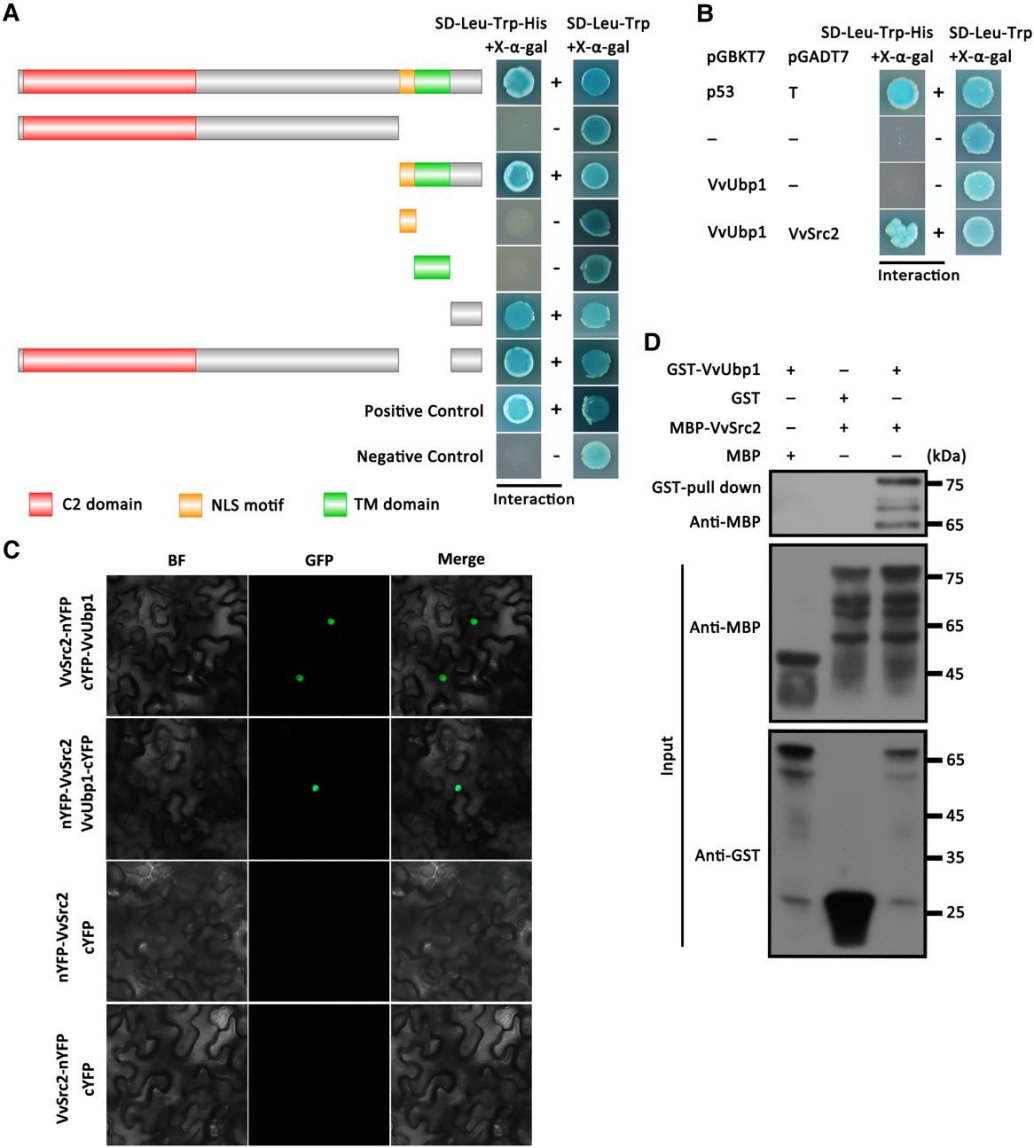
VvSrc2 interacted with VvUbp1. **A)** Investigation of the transcriptional autoactivation activity of VvSrc2 by yeast two-hybrid. A series of VvSrc2 truncations was assayed for the ability to permit the growth of yeast transformants on auxotrophic media lacking leucine(-Leu), tryptophan(-Trp), and histidine(-His) and the development of blue color in the presence of X-α-gal. The obtained results uncovered that the C-terminus region of VvSrc2 (VvSrc2^272-291^) was necessary and sufficient for its transcriptional autoactivation activity. Yeast cells expressing the empty vector *pGADT7* with empty vector *pGBKT7* were used as negative controls. The interaction between *pGADT7-T* with *pGBKT7-53* was used as the positive control. The symbols + and − marked the presence of interaction and absence of interaction, respectively. SD, synthetic dropout. **B)** Interaction confirmation of VvSrc2 and VvUbp1 by yeast two-hybrid. The positive and negative controls were the same as those mentioned in **(A)**. **C)** Interaction confirmation of VvSrc2 and VvUbp1 using a bimolecular fluorescence complementation (BiFC) assay in *N. benthamiana*. The VvSrc2 and VvUpb1 were fused to the N-terminus and C-terminus of the YFP epitope tag, respectively. Fluorescence signal was detected with a microscope 2 d post-infiltration (dpi). YFP, yellow fluorescent protein. **D)** Interaction validation of VvSrc2 with VvUbp1 using MBP pull-down experiments. The recombinant proteins GST-LtLysM2 and MBP-VvSrc2 purified from *E. coli* were subjected to GST pull-down analyses followed by immunoblot analyses. GST, glutathione S-transferase. MBP, maltose binding protein.

Because of the elevated nuclear accumulation of VvSrc2 in the presence of LtLysM2, we tested to decipher its molecular roles in relation to the cell nucleus. First, we performed a stringent yeast two-hybrid screening against the cDNA prey library derived from grapevine tissues to identify the host interactors of VvSrc2. It was found that yeast cells expressing the bait construct of VvSrc2 displayed a transcriptional autoactivation activity, and therefore, we generated a series of truncations to determine the key region that was essential for the activity. The obtained results showed that only yeast transformants expressing the C-terminus region (VvSrc2^272-291^) or other truncations containing this domain enabled the growth of yeast cell on selective medium plate and displayed α-galactosidase activities, suggesting this region was required for its autoactivation activity (Fig. 5A). Additionally, as the VvSrc2 protein contained a predicted transmembrane domain (spanning residues 248 to 272), and therefore, an N-terminal moiety deletion of the two domains (residues 248 to 272 and 272 to 291) was used as the bait to capture the interactors of VvSrc2 by a yeast two-hybrid screening against the prey library described above. Totally, we detected 4 proteins that potentially associated with VvSrc2 (Supplementary Table S4), including a predicted RNA-binding protein VvUbp1 featuring 2 RRM domains defined by the Pfam program. Because the VvSrc2/VvUbp1 pair has not been studied so far, and the wheat homolog of VvUbp1 was documented to be involved in mediating plant immunity in previous report (Li et al. 2022), we decided to investigate this interaction hereafter.

Following the confirmation by yeast 2 hybrid (Fig. 5B), we adopted a bimolecular fluorescence complementation (BiFC) approach to validate the association between the 2 proteins. For the BiFC assays, VvSrc2 fused to the N-terminal fragment of YFP (yellow fluorescent protein) was co-expressed with VvUbp1 fused to the C-terminal fragment of YFP. The fluorescence detected by microscopy validated a direct interaction between VvUbp1 and VvSrc2 in vivo (Fig. 5C).

To offer more persuasive proofs to evidence of the interaction, we performed an in vitro pull-down experiment to test the interaction between VvSrc2 and VvUbp1. The recombinant MBP-VvSrc2 protein was purified from *E. coli* with MBP magnetic beads and incubated with GST-VvUbp1 proteins purified with glutathione agarose. Protein immunoblotting analyses showed that GST-VvUbp1 was pulled down by MBP-VvSrc2 protein, illustrating a physical association between VvUbp1 and VvSrc2 (Fig. 5D).

### VvSrc2 contributed to the cell death triggered by VvUbp1

**Figure 6.**
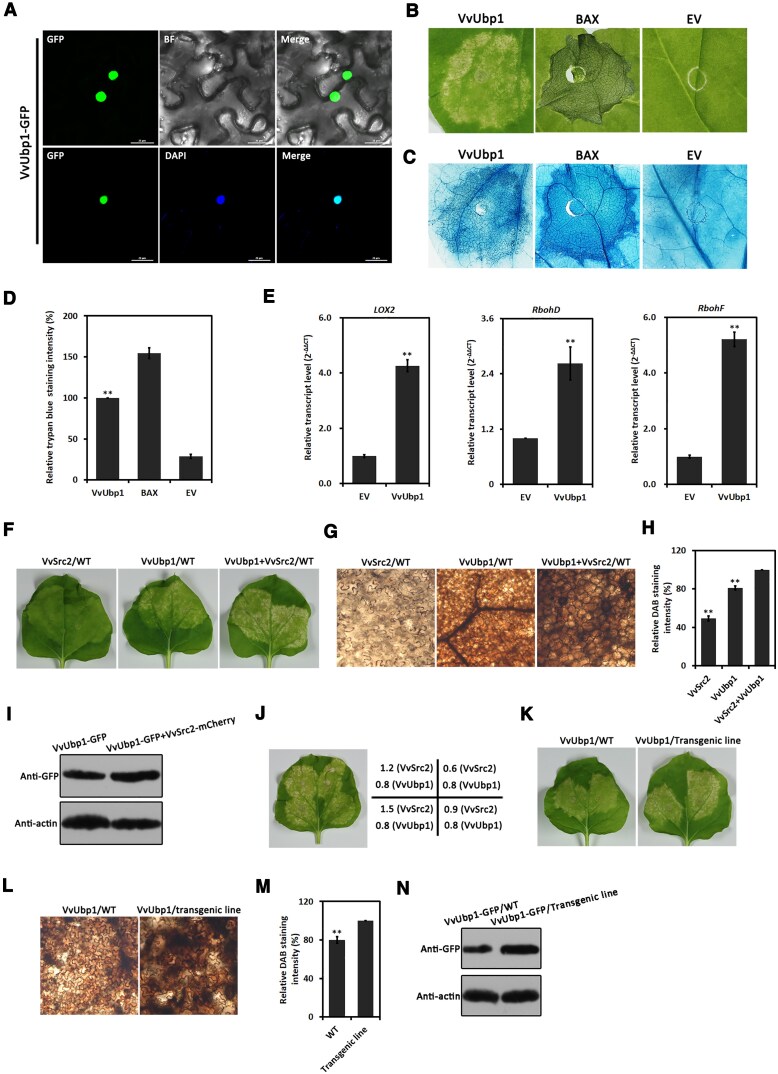
VvSrc2 contributed to the cell death evoked by VvUbp1. **A)** Subcellular localization detection of VvUbp1 in plants. *Nicotiana benthamiana* expressing VvUbp1-GFP fusion protein displayed strong intensity of green fluorescence that overlapped with the blue fluorescence from the cell nucleus stained by DAPI agent. BF, bright field. **B, C)** Investigation of the elicitor activity of VvUbp1. Cell death of *N. benthamiana* transiently expressing VvUbp1 was detected 5 d post-agroinfiltration **(B)**, followed by trypan blue staining **(C)**. **D)** Measurement of trypan blue staining intensity. Three biological replicates are performed and the presented data are means ± Se. Significant differences were evaluated using the one-way analysis of variance (ANOVA) and least significant difference (LSD) tests (**α = 0.01). **E)** Transcription examination of ROS metabolism-related genes by qRT-PCT. The experiment and data analyses were performed with similar methods as shown in **(A)**. **F, G)** Investigation of cell death activated by VvSrc2, VvUbp1, and VvSrc2/VvUbp1 pair. Cell death of *N. benthamiana* transiently expressing VvSrc2, VvUbp1, and VvSrc2/VvUbp1 pair was detected 5 d post-agroinfiltration **(F)**, followed by 3,3-diaminobenzidine (DAB) staining **(G)**. **H)** Intensity measurement of DAB staining. The presented data are means ± Se of 3 replicates. Significant differences were evaluated using the one-way analysis of variance (ANOVA) and least significant difference (LSD) tests (** *α* = 0.01). The leave samples were the same as those mentioned in **(F)**. **I)** Immunoblot analyses of VvUbp1 in the presence/absence of VvSrc2. **J)** Investigation of cell death activated by VvUbp1 with gradually increasing concentrations of VvSrc2. The concentration of adjusted *A. tumefaciens* was denoted in the right panel. **K, L)** Investigation of cell death activated by VvUbp1 on wild type (WT) and *VvSrc2*-transgenic line. Cell death of *N. benthamiana* transiently expressing VvSrc2 was detected 5 d post-agroinfiltration **(K)**, followed by 3,3-diaminobenzidine (DAB) staining **(L)**. **M)** Intensity measurement of DAB staining. The presented data are means ± Se of 3 replicates. Significant differences were evaluated using the one-way analysis of variance (ANOVA) and least significant difference (LSD) tests (** *α* = 0.01). The leave samples were the same as those mentioned in **(K)**. **N)** Immunoblot analyses of VvUbp1 in wild type (WT) and *VvSrc2-transgenic line*. *Nicotiana benthamiana* transiently expressing VvUbp1 were collected 2 d post-agroinfiltration.

As VvUbp1 was predicted to be an RNA-binding protein, we thereby detected the spatial distribution of this protein by confocal microscopy. A VvUbp1-GFP fusion construct was engineered and transiently expressed in *N. benthamiana* by the *A. tumefaciens*-mediated transformation. Notably, it was observed that green fluorescence from VvUbp1-GFP was accumulated in the nucleus when expressed in *N. benthamiana* (Fig. 6A). Additionally, the fluorescence signal was overlapped with the blue fluorescence emitted by the cell nucleus stained by the DAPI agent, confirming that VvUbp1 was a nucleus-localized protein in planta.

In terms of VvUbp1, previous research reported its homolog, TaUBA2C in wheat (sharing 42% identities in amino acids with VvUbp1), was able to inhibit the CWMV infection through recruiting the pre-mRNA of immunity-related genes to induce cell death and H_2_O_2_ production (Li et al. 2022). Here, we sought to investigate whether the VvUbp1 harbored similar activity and, therefore, transiently expressed the VvUbp1-GFP fusion protein in *N. benthamiana* leaves through agroinfiltration. *Agrobacterium tumefaciens* carrying the proapoptotic protein BAX and an empty vector (EV) were used as positive and negative controls, respectively. Notably, a type of suicidal cell death was presented in *N. benthamiana* leaves expressing VvUbp1-GFP protein; the intensity of cell death, however, was weaker than that of positive control, which was further verified by trypan blue staining (Fig. 6B to D), suggesting that, similar to its orthologs TaUBA2C, VvUbp1 also functioned as a cell death activator in plant. Additionally, we assessed the effect of VvUbp1 on the expression of defense and H_2_O_2_ biogenesis-related genes. The resulting data showed that the transcript accumulation of these genes was evidently elevated in the *N. benthamiana* expressing VvUbp1 relative to that of the control (Fig. 6E), implying that VvUbp1 may serve as an immunity mediator through mediating the expression of ROS burst-related genes.

As an interactor of VvUbp1, we tested to examine whether VvSrc2 had an effect on the elicitor activity of VvUbp1. Therefore, we transiently expressed VvUbp1, VvSrc2, and VvUbp1/VvSrc2 pair in *N. benthamiana* and assessed the programmed cell death lesions 7 d post-agroinfiltration (dpi). It is of note that, expression of VvUbp1 triggered relatively mild cell death, in terms of the cell death provoked by VvUbp1/VvSrc2 pair (Fig. 6F). Additionally, after the treatment with DAB agent, *N. benthamiana* leaves expressing the VvUbp1/VvSrc2 pair displayed a darker-brown color, indicative of ROS biogenesis, relative to that of VvUbp1 by microscopy; the expression of VvSrc2, however, elicited a light yellow-brown color, suggesting that VvSrc2 may serve as a positive helper in VvUbp1 mediated-ROS burst during infection (Fig. 6G and H). Furthermore, immunoblot analyses revealed that the protein amount of VvUbp1 was elevated in the presence of VvSrc2, which further confirmed the results mentioned above (Fig. 6I). Additionally, simultaneous expression of VvUbp1, together with gradually increasing concentrations of VvSrc2, further confirmed the above results (Fig. 6J). To further validate the above results, the VvUbp1 protein was simultaneously expressed in wild-type and *VvSrc2*-transgenic line by agroinfiltration. Following treatment with the DAB agent, a relatively darker brown color was presented in the *VvSrc2*-transgenic line (Fig. 6K to M), suggesting that VvSrc2 was beneficial for ROS accumulation evoked by VvUbp1. The results were also verified by an immunoblot analysis, which showed that, in terms of VvUbp1, a higher level of protein accumulation was detected in the *VvSrc2*-transgenic line than that of the wild type (Fig. 6N).

**Figure 7.**
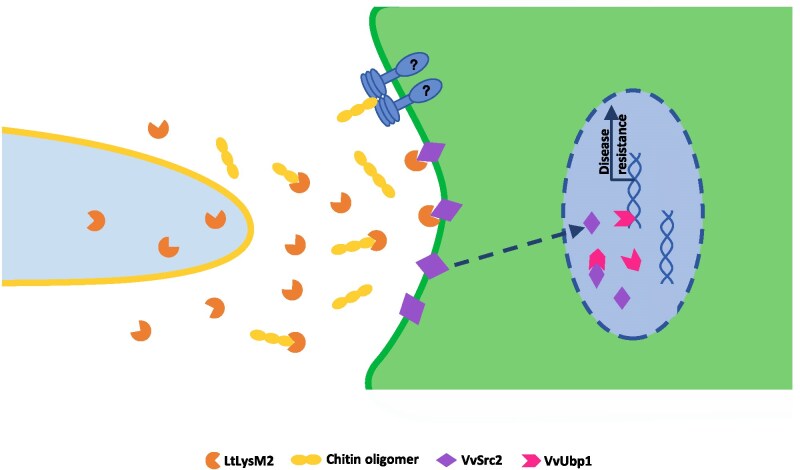
A proposed working model of LtLysM2. During infection, fungal chitin may be perceived by an undisclosed receptor in grapevine, resulting in an immune response; the chitin-triggered immunity, however, is suppressed by the effector LtLysM2 secreted by *L. theobromae*. Interestingly, LtLysM2 interacts with grapevine protein VvSrc2 and promotes the nuclear accumulation of VvSrc2; The VvSrc2 protein, conversely, inhibits the immunity-suppressive function of LtLysM2, possibly by competing with chitin to bind to LtLysM2 and an occupancy effect. In addition, the grapevine protein VvSrc2 associates with a nuclear-localized protein VvUbp1 and the overexpression of *VvSrc2* contributes to the protein accumulation of VvUbp1 and the programmed cell death evoked by VvUbp1. The VvUbp1, a putative RNA-binding protein homologous to the *Triticum aestivum* TaUBA2C, possibly activates host immunity through recruiting the pre-mRNA of ROS burst-related genes and mediating their expression.

Based on these results, it is proposed that during infection, the chitin-triggered immunity is suppressed by the effector LtLysM2 secreted by *L. theobromae*. Additionally, LtLysM2 interacts with grapevine protein VvSrc2 and promotes the nuclear accumulation of VvSrc2; the VvSrc2 protein, on the other hand, inhibits the immunity-suppressive function of LtLysM2. Interestingly, the grapevine protein VvSrc2 associates with a nuclear-localized protein VvUbp1 and overexpression of *VvSrc2* contributes to the programmed cell death evoked by VvUbp1. The VvUbp1, a putative RNA-binding protein homologous to the *Triticum aestivum* TaUBA2C (Li et al. 2022), possibly activates host immunity through recruiting the pre-mRNA of ROS burst-related genes and mediating their expression (Fig. 7).

## Discussion

The LysM proteins were widely identified in various organisms, including plants, fungi, and bacteria (de Jonge et al. 2010; Shimizu et al. 2010; Kombrink and Thomma 2013; Sánchez-Vallet et al. 2013; Tanaka et al. 2013; Rovenich et al. 2014; Akcapinar et al. 2015; Kombrink et al. 2017). Although the virulence/avirulence functions of LysM effectors are widely characterized in grain and foliar pathogens, including *C. fulvum* (de Jonge et al. 2010), *M. graminicola* (Marshall et al. 2011), *M. oryzae* (Mentlak et al. 2012), and *C. higginsianum* (Takahara et al. 2016), the biological functions of LysM effectors are barely investigated in woody plant pathogens. In this study, we characterized a LysM effector, LtLysM2, in the plant opportunistic pathogen *L. theobromae*, which has been reported to be able to invade a wide range of woody plants (Yan et al. 2018). Our objective is to understand how the LtLysM2 protein regulates plant immunity and whether this protein has an additional role beyond its previously documented function. Here, our findings point to a working model in which the effector LtLysM2 interacts with grapevine protein VvSrc2 and promotes the nuclear accumulation of VvSrc2; additionally, the grapevine protein VvSrc2 associates with a putative RNA-binding protein VvUbp1 and overexpression of *VvSrc2* contributes to the programmed cell death evoked by VvUbp1.

In terms of LysM proteins, previously reported effectors, including Avr4 (van den Burg et al. 2006), Ecp6 (de Jonge et al. 2010), Slp1 (Mentlak et al. 2012), ChELP1 and ChELP2 (Takahara et al. 2016), were able to suppress the chitin-triggered immunity or protect the fungal hyphae against plant chitinases. Our understanding of the biological roles of LysM effectors remains relatively limited. Here, we directly linked the secreted effector protein LtLysM2 with a host protein VvSrc2 that is located at the cell membrane and nucleus. As an interactor of LtLysM2, the VvSrc2 protein was able to inhibit the immunity-suppressive function of LtLysM2. The underlying mechanism of the inhibition is unrevealed in the study. However, according to the established interaction between LtLysM2 with VvSrc2, and the chitin-binding ability of LtLysM2, we hypothesize that VvSrc2 possibly interferes with the association of LtLysM2 with chitin through a competitive occupancy effect to influence the molecular roles of chitin and mediate plant immunity. Whether the LtLysM2, VvSrc2, and chitin molecule could form a ternary complex remains unknown. Further structural investigations are needed to reveal the answer to the hypothesis. Besides, although the membrane-localized chitin receptor in model plants such as rice (Shimizu et al. 2010) and *Arabidopsis thaliana* (Petutschnig et al. 2010) has been well documented, perception of chitin oligosaccharides in grapevine is not yet reported. As a membrane component, whether VvSrc2 associates with the chitin receptor and responds to the recognition of chitin by its receptor opens an interesting question in the future.

More importantly, we found that VvSrc2 interacted with the secreted effector LtLysM2 and a putative RNA-binding protein VvUbp1. The association among the ternary components implicates that VvSrc2 may function as a signal transducer to recognize the effector and subsequently transfer upstream signal events to nuclear components to activate corresponding responses, which can be supported indirectly by the experimental results that the nuclear amount of VvSrc2 protein is increased in the presence of LtLysM2. In terms of the trafficking mechanism, it was unrevealed in the current study. A documented Src2 homolog, AtSrc2, identified from *Arabidopsis thaliana,* was confirmed to traffic from the endoplasmic reticulum (ER) to the vacuole by an intermediate organelle, named precursor-accumulating (PAC) vesicles, by budding directly from ER (Oufattole et al. 2005). Possibly, the VvSrc2 protein may be trafficked into the nucleus in a similar pathway, and the assumption, however, opened an interesting question for future study. With regard to the VvUbp1, its wheat homolog TaUBA2C (sharing 42% identities in amino acids with VvUbp1) was confirmed to be capable of recruiting the pre-mRNA of *TaNPR1*, *TaPR1,* and *TaRbohD* to induce cell death and H_2_O_2_ production (Li et al. 2022). Similar to TaUBA2C, VvUbp1 also caused a type of suicidal cell death, and the intensity of VvUbp1-triggered cell death was enhanced by VvSrc2; transient expression of VvSrc2 alone, however, could not trigger the cell death symptom, suggesting that VvSrc2 possibly served as a helper in VvUbp1-triggered plant defense response. Immunoblot examination revealed that the protein accumulation of VvUbp1 was elevated in the presence of VvSrc2, indicating that the enhanced cell death by VvSrc2 may be a result of the elevated protein accumulation of VvUbp1. Of course, more complicated regulatory mechanisms may exist between VvSrc2 and VvUbp1, which is beyond the scope of the study, and further work will continue to answer this interesting question.

In summary, our data indicate that VvSrc2 functions as an intermediary regulator to transform extracellular signal events evoked by LyLysM2 to nuclear components to mount the interactive outcome of *L. theobromae* with the host plant. This study reveals a pathway for LysM effector proteins to mediate plant defence responses. Further exploration will be required to decipher the underlying regulatory mechanism between VvSrc2 and VvUbp1.

## Materials and methods

### Bacterial strains, plant materials, and growth conditions

The *L. theobromae* wild-type strain CSS-01s, overexpression and silencing transformants of *LtLysM2* were cultured in complete medium (6 g yeast extract, 3 g casein acid hydrolysate, 3 g casein enzymatic hydrolysate, 10 g sucrose, and 16 g agar per liter). The *Agrobacterium tumefaciens* strain GV3101 was cultured in LB medium (5 g yeast extract, 10 g tryptone, 10 g NaCl, and 16 g agar per liter). The yeast strain YTK12 and its transformants were cultured in YPDA (1% yeast extract, 2% peptone, 2% glucose, 0.003% adenine hemisulfate, and 2% agar for plate), CMD-W (0.67% yeast nitrogen without amino acids, 0.075% tryptophan dropout supplement, 2% sucrose, 0.1% glucose, and 2% agar), and YPRAA (1% yeast extract, 2% peptone, 2% raffinose, 2 μg/mL antimycin A, and 2% agar) media. *N. benthamiana* was grown in a chamber at 25 °C under 14 h:10 h light:dark conditions.

### Targeted gene overexpression and silencing of *LtLysM2*

To construct the overexpression vector of the *LtLysM2* gene, the ORFs of *LtLysM2* were amplified from cDNA of *L. theobromae* with primer pair LtLysM2OE-f/LtLysM2OE-r, and were ligated into vector *pKSNTP* as the overexpression vector, referred to as *pKSNTP:LtLysM2.* Subsequently, the fusion construct *pKSNTP:LtLysM2* was digested with the restriction enzyme *Not* I and transformed into *L. theobromae* protoplast using the PEG-mediated transformation method as described by Yang et al. (2010). In terms of the silencing of *LtLysM2*, the construction of the fusion vector and PEG-mediated transformation were performed with similar methods used for the overexpression of *LtLysM2*. The resultant transformants were isolated and tested for their resistance to neomycin and were screened by RT-qPCR assays. The positive transformants were selected for subsequent phenotypic analyses.

### Pathogenicity test

The virulence of *L. theobromae* wild type strain CSS-01s, *LtLysM2* overexpression and silencing transformants were examined by inoculating 1-d-old mycelial plugs (5 mm in diameter) on 1-yr-old wound shoots of susceptible grapevine cultivar *Vitis vinifera* L. “Summer Black,” and then the inoculated grapevine shoots were contained inside a chamber under constant humidity and temperature. The incidence degree was quantified by measuring the lesion length of diseased grapevine shoots at 72 h post-inoculation (hpi). The experiments were performed independently with 3 replicates.

### RNA isolation and gene expression analyses

Pathogenicity test was performed according to the method described above. The inoculated grapevine tissues were harvested at different time points, including 12, 24, 48, and 72 hpi, and used for subsequent RNA isolation. RNA was extracted using an RNA isolation kit, according to the manufacturer's instructions (Aidlab Biotech, Beijing, China). The cDNA was synthesized using the TransScript One-Step gDNA Removal and cDNA Synthesis SuperMix kit (TransGen Biotech, Beijing, China). qRT-PCR was performed using the ABI 7500 Real Time system (Applied Biosystems, Waltham, USA) with 2×RealStar Green Fast Mixture with ROX II. The qRT-PCR assays were conducted in a 16 μL final volume consisting of 1.0 μL cDNA, 0.2 μM primer, 8 μL RealStar Green Fast Mixture with ROX II, and 6.4 μL sterile water. The amplification protocol is as follows: 2 min for denaturation at 95 °C, followed by 40 cycles of 95 °C for 15 s and 60 °C for 30 s. The stable *actin* gene was amplified as an internal control. Expression data were normalized by *actin* gene and calibrated against the transcript level of mycelia. The relative abundance of transcripts was calculated using the 2^−ΔΔCT^ method (Livak and Schmittgen 2001). The experiments were repeated 2 times independently with 3 replicates each time. All of the primers presented in Supplementary Table S5 are synthesized by Biomed (Beijing, China).

### *Agrobacterium tumefaciens*-mediated transient expression

The full-length and truncated moieties of LtLysM2 were amplified using the I-5TM High-Fidelity DNA Polymerase (TSINGKE Biological Technology, Beijing, China) with primers listed in Supplementary Table S5, and cloned into an expression vector driven by the cauliflower mosaic virus 35S promoter 35 s for subsequent transient expression. The fusion construct was transformed into *A. tumefaciens* GV3101 according to the manufacture's manual. The overnight cultured *A. tumefaciens* transformants were harvested and washed with sterile double-distilled H_2_O 3 times, and subsequently resuspended in infiltration buffer (10 mm MES, pH 5.7, 10 mm MgCl_2_, and 150 μM acetosyringone) with an optical density value equal to 0.6 at 600 nm. After standing at room temperature for 3 h, the *A. tumefaciens* cultures were infiltrated into 4-wk-old *N. benthamiana* leaves using needleless syringes. *A. tumefaciens* strains expressing the proapoptotic protein BAX were used as the positive control. The infiltration buffer and *A. tumefaciens* strain expressing the empty vector (EV) were used as negative controls.

### Functional confirmation of predicted SP

Functional validation of predicted LtLysM2 SP was performed using a yeast SP trap system. The yeast SP trap vector, *pSUC2T7M13ORI* (*pSUC2*), contains a truncated invertase-encoding gene lacking both its initiating methionine codon and SP sequence (Jacobs et al. 1997; Fang et al. 2016). The encoding sequence of LtLysM2 SP was amplified with primer pair LtLysM2SP-f/LtLysM2SP-r and then ligated into *pSUC2* vector with *Eco* RI/*Xho* I restriction enzymes. Subsequently, the fusion construct *pSUC2:LtLysM2* was transformed into yeast strain YTK12 using a Yeastmaker yeast transformation system 2 kit, according to the manufacturer's instructions (Clontech, CA, USA). The fusion constructs carrying the N-terminal sequences of *P. sojae* Avr1b and *M. oryzae* Mg87 were used as the positive and negative controls, respectively. The resultant transformants were streaked on the CMD-W and YPRAA plates for invertase selection.

### Yeast two-hybrid assay

The yeast two-hybrid assays were conducted with the Yeastmaker yeast transformation system 2 kit, according to the Yeast Protocols Handbook (Clontech, CA, USA). VvSrc2 ORFs and its truncated moieties were amplified with primers presented in Supplementary Table S5 and cloned into *pGADT7*, referred to as prey constructs. The VvChi4 ORFs with its truncated forms were ligated into *pGADT7* with a similar method. The LtLysM2 ORFs were amplified and cloned into *pGBKT7* as the bait construct. Each prey and bait construct pair was co-transformed into the yeast strain AH109. The resulting transformants were assayed for growth on both SD-Leu-Trp and SD-Leu-Trp-His media, and for X-*α*-galactosidase activities.

### Pull-down assays

The cDNA sequences of LtLysM2 were amplified with primer pair LtLysM2GST-f/LtLysM2GST-r (Supplementary Table S5), and subcloned into the protein expression vector *pGEX-4T-1*. The cDNA sequences of VvSrc2 were amplified with primer pair VvSrc2MBP-f/VvSrc2MBP-r, and ligated into another expression vector, *pMAl-C4X.* The cDNA sequences of *VvUbp1* were amplified with primer pair VvUbp1GST-f/VvUbp1GST-r, and cloned into *pGEX-4T-1* vector using a method similar to that used for VvSrc2. All of the fusion vectors were transformed into *E. coli* BL21 for further recombinant protein expression, respectively. Protein purification and MBP pull-down assays were performed according to the method described by Liu et al. (2011).

### BIFC assays

For split-YFP assays, leaves of 4-wk-old *N. benthamiana* were infiltrated with agrobacteria carrying the N-terminal fragment fused to VvSrc2 and the C-terminal fragment fused to LtLysM2 or VvUbp1. For each combination pair, agrobacteria were infiltrated at an OD600 of 0.8. Leaf pieces were imaged on a Zeiss 710 confocal microscope 2 d after agroinfiltration at an excitation wavelength of 514 nm and collection wavelength of 525 to 560 nm.

### Laser-scanning confocal microscopy

In terms of fluorescence detection, GFP-epitope and mCherry-epitope tagged proteins were transiently expressed in *N. benthamiana* by agroinfiltration. Patches of infiltrated *N. benthamiana* leaves were cut and mounted in water and analyzed on a Zeiss 710 confocal microscope at an excitation wavelength of 488 nm (for green fluorescence detection) or 561 nm (for red fluorescence detection).

### Polysaccharide affinity precipitation assay

The affinity of LtLysM2 for various polysaccharides was determined by incubating 50 μg/mL of LtLysM2 with 5 mg chitin beads (New England Biolabs) and chitin from shrimp shells (Sigma–Aldrich) as previously described (van den Burg et al. 2006). The incubation was performed at 4 °C on a rocking platform in a final volume of 600 μL buffer containing 20 mm Tris/HCl, pH 8.0, and 150 mm NaCl. After 12 h of gentle rocking at 4 °C, the insoluble fraction was pelleted by centrifugation (5 min, 13,000 × *g*). The insoluble fraction was washed 3 with incubation buffer to remove the unbound proteins and boiled in 100 μL of 1% SDS solution before being examined by SDS–PAGE followed by Coomassie brilliant blue staining.

### DAB staining

Host-derived ROS accumulation was detected by staining with 3, 3′-diaminobenzidine (DAB) solution as described by Ding et al. (2010). Briefly, the agroinfiltrated *N. benthamiana* leaves were incubated in 1 mg/mL DAB solution (pH 3.8) for 24 h, followed by destaining in a mixture composed of ethanol (80%), distilled water (10%), and glacial acetic acid (10%) overnight. The relative intensity of DAB staining was calculated using the ImageJ software.

### Trypan blue staining

For trypan blue staining, the agroinfiltrated *N. benthamiana* leaves were boiled and stained in trypan blue solution (Sigma–Aldrich), followed by destaining in chloral hydrate solution (2.5 g/mL) overnight to visualize dead cell symptoms. The relative intensity of trypan blue staining was calculated using the ImageJ software.

### Accession numbers

Sequence data from this article can be found in the GenBank/EMBL data libraries. LtLysM2 (XM_035514980.1), VvSrc2 (XM_002272868.5), VvUbp1 (XM_002278566.4), VvChi4 (NM_001281146.1)_l.

## Supplementary Material

kiaf608_Supplementary_Data

## Data Availability

The data underlying this article will be shared on reasonable request to the corresponding author.

## Supplementary data

The following materials are available in the online version of this article.

**Supplementary Figure S1.** The LysM proteins are distributed widely across fungal taxa.

**Supplementary Figure S2.** LtLysM2 interacts with VvChi4.

**Supplementary Figure S3.** Schematic illustration of VvSrc2.

**Supplementary Figure S4.** Subcellular localization of truncated VvSrc2 in *N. benthamiana*.

**Supplementary Figure S5.** Statistical analyses of differentially expressed genes (DEGs) between the wild type and the *VvSrc2*-transgenic line without pathogen infection.

**Supplementary Figure S6.** Statistical analyses of differentially expressed genes (DEGs) between the wild type and the *VvSrc2*-transgenic line infected by *L. theobromae*.

**Supplementary Table S1.** Systematic identification of LysM proteins in *L. theobromae*.

**Supplementary Table S2.** The potential interacting targets of LtLysM2 obtained by cDNA library screening.

**Supplementary Table S3.** Gene Ontology classification of enriched DEGs identified from pairwise comparison between the wild type and the *VvSrc2*-transgenic line infected by *L. theobromae*.

**Supplementary Table S4.** The potential interacting targets of VvSrc2 obtained through cDNA library screening.

**Supplementary Table S5.** Primers used in this study.

## Dive Curated Terms

The following phenotypic, genotypic, and functional terms are of significance to the work described in this paper:

BAP1 Gramene: AT3G61190
BAP1 Araport: AT3G61190

Bap2 Gramene: AT2G45760
Bap2 Araport: AT2G45760

CRN Gramene: AT5G13290
CRN Araport: AT5G13290

DAPI CHEBI: CHEBI:51231

protein CHEBI: CHEBI:36080
