# Supplementary material for: Grapevine protein Src2 mediates plant disease resistance during Lasiodiplodia theobromae infection
Source: Plant Physiol. 2025 Nov 24;199(4):kiaf608. doi: 10.1093/plphys/kiaf608 (PMC12684713; doi:10.1093/plphys/kiaf608)
Supplement: kiaf608_Supplementary_Data [file kiaf608_supplementary_data.zip › 20251116 Supplementary Data.pdf]

Supplementary Figure S1. The LysM proteins are distributed widely across fungal taxa. (**A**) Phylogenetic analyses of LysM proteins in five different fungal species (*Magnaporthe oryzae*, *Cladosporium fulvum*, *Colletotrichum higginsianum*, *Mycosphaerella graminicola*, and *Lasiodiplodia theobromae*). The amino acid sequences of the LysM proteins from different fungal species were sourced from the NCBI database and then used to generate a phylogenetic tree using MEGA4, with the neighbor-joining method and 1,000 replicates. Bootstrap support values (%) are indicated at the nodes. The scale bar is indicative of 0.2 changes per amino acid position. (**B**) Schematic illustration of LtLysM2. SP, signal peptide; LysM, LysM domain.

Supplementary Figure S2. LtLysM2 interacts with VvChi4. (**A**) Confirmation of the interaction between LtLysM2 and VvChi4 by yeast two-hybrid assay. The positive and negative controls were the same as those mentioned in (Figure 2A). The symbols + and – mark the presence of interaction and absence of interaction, respectively. SD, synthetic dropout. (**B**) Validation of the interaction between tLysM2 and VvChi4 using MBP pull-down experiments. The recombinant proteins GST-LtLysM2 and MBP-VvChi4 purified from *E. coli* were subjected to MBP pull-down analyses. The interacting protein was detected via immunoblotting. GST, Glutathione S-Transferase. MBP, Maltose Binding Protein.

Supplementary Figure S3. Schematic illustration of VvSrc2.

Supplementary Figure S4. Subcellular localization of truncated VvSrc2 in *N. benthamiana*. (A) Observation of the subcellular localization for a series of VvSrc2 truncations in *N. benthamiana*. Comparatively, deletion of the transmembrane domain of VvSrc2 (ΔTM) had an unobservable effect on the subcellular localization of VvSrc2; by contrast, VvSrc2 with a mutated NLS motif (nls) accumulated in the nucleus. VvSrc2 lacking the C2 domain and transmembrane domain (ΔTM+ΔC2) simultaneously or lacking the C2 domain only (ΔC2) mainly accumulated in the nucleus and also gathered together to form small clumps. (**B**) Expression confirmation of the proteins mentioned in (A) by immunoblotting.

Supplementary Figure S5. Statistical analyses of differentially expressed genes (DEGs) between the wild type and the *VvSrc2*-transgenic line without pathogen infection.

Supplementary Figure S6. Statistical analyses of differentially expressed genes (DEGs) between the wild type and the *VvSrc2*-transgenic line infected by *L. theobromae*.

Supplementary Table S1. Systematic identification of LysM proteins in *L. theobromae*. LysM proteins were identified by BLASTp using the amino acid sequence of *Cladosporium fulvum* Ecp6 as the query subject. The number of LysM domains of six *L. theobromae* proteins was predicted using the Pfam program.

Supplementary Table S2. The potential interacting targets of LtLysM2 obtained through cDNA library screening. The LtLysM2 bait vector was transformed into yeast strain Y2HGold to fish for the candidates that interacted with LtLysM2. Functions of interacting candidates were analyzed using the NCBI blast program, and the subcellular localizations were predicted by Psort II.

Supplementary Table S3. Gene Ontology classification of enriched DEGs identified from pairwise comparison between the wild type and the *VvSrc2*-transgenic line infected by *L. theobromae*.

Supplementary Table S4. The potential interacting targets of VvSrc2 obtained through cDNA library screening. The VvSrc2 bait vector was transformed into yeast strain Y2HGold to capture the candidates that potentially interacted with VvSrc2. Functions of interacting candidates were predicted using the NCBI blast program.

Supplementary Table S5. Primers used in this study.

Supplementary Figure S1. The LysM proteins are distributed widely across fungal taxa.

A

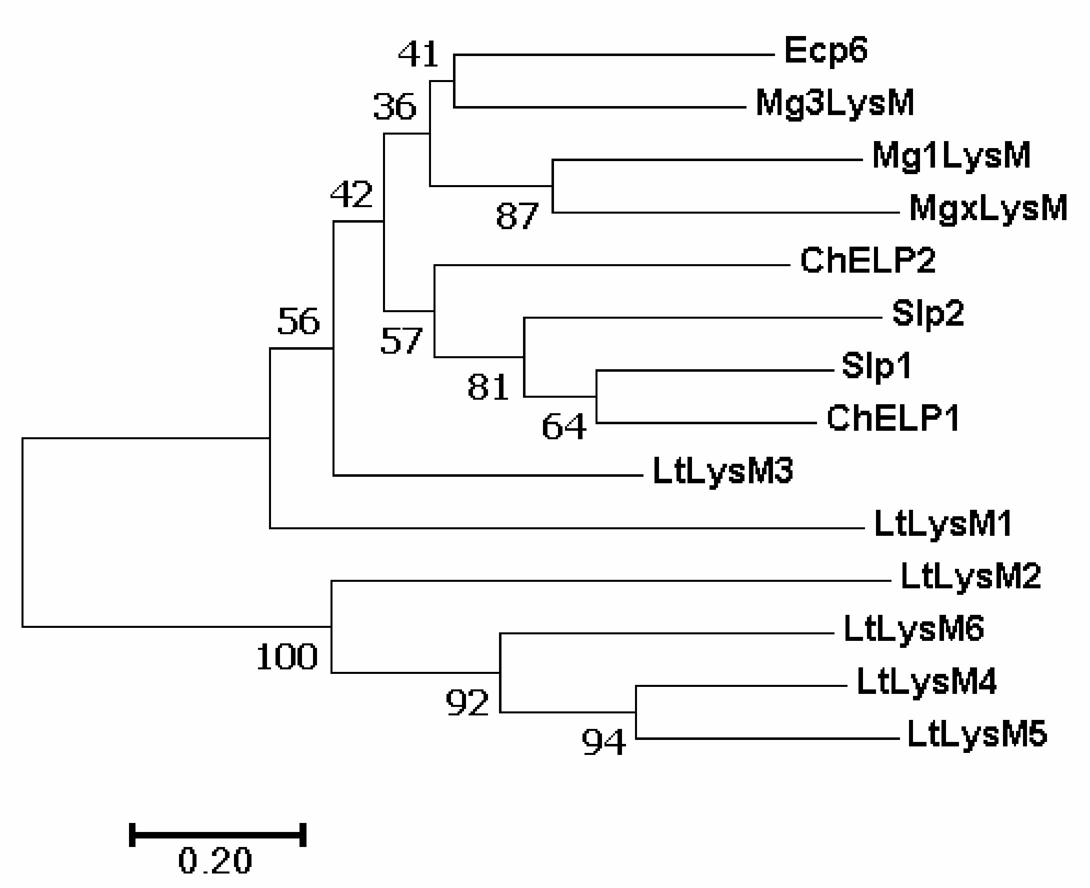

B

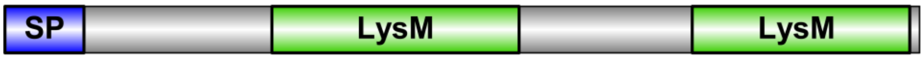

SP: signal peptide LysM: LysM domain

Supplementary Figure S2. LtLysM2 interacts with VvChi4.

A

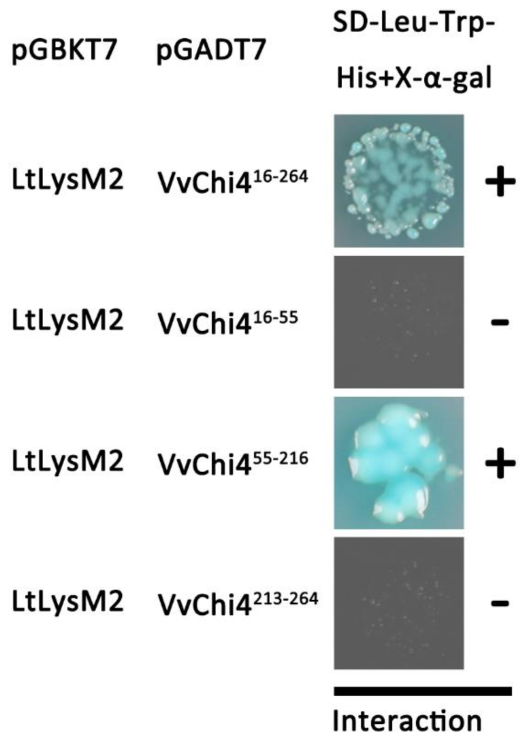

B

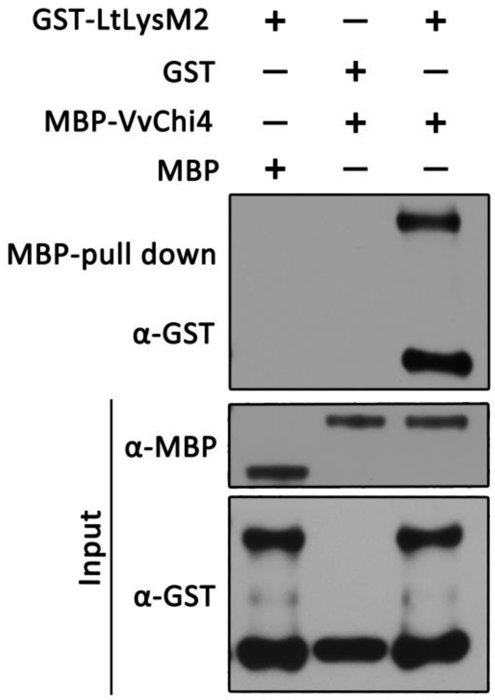

Supplementary Figure S3. Schematic illustration of VvSrc2.

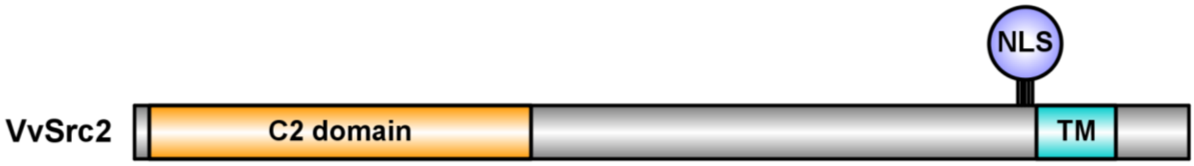

NLS: nuclear localization signal   TM: Transmembrane domain

Supplementary Figure S4. Subcellular localization of truncated VvSrc2 in *N. benthamiana*.

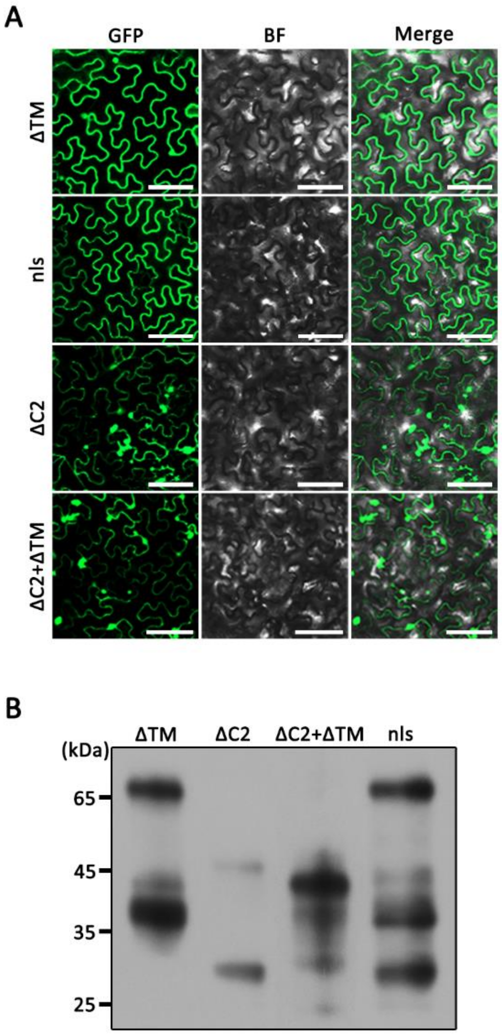

Supplementary Figure S5. Statistical analyses of differentially expressed genes (DEGs) between the wild type and the *VvSrc2*-transgenic line without pathogen infection.

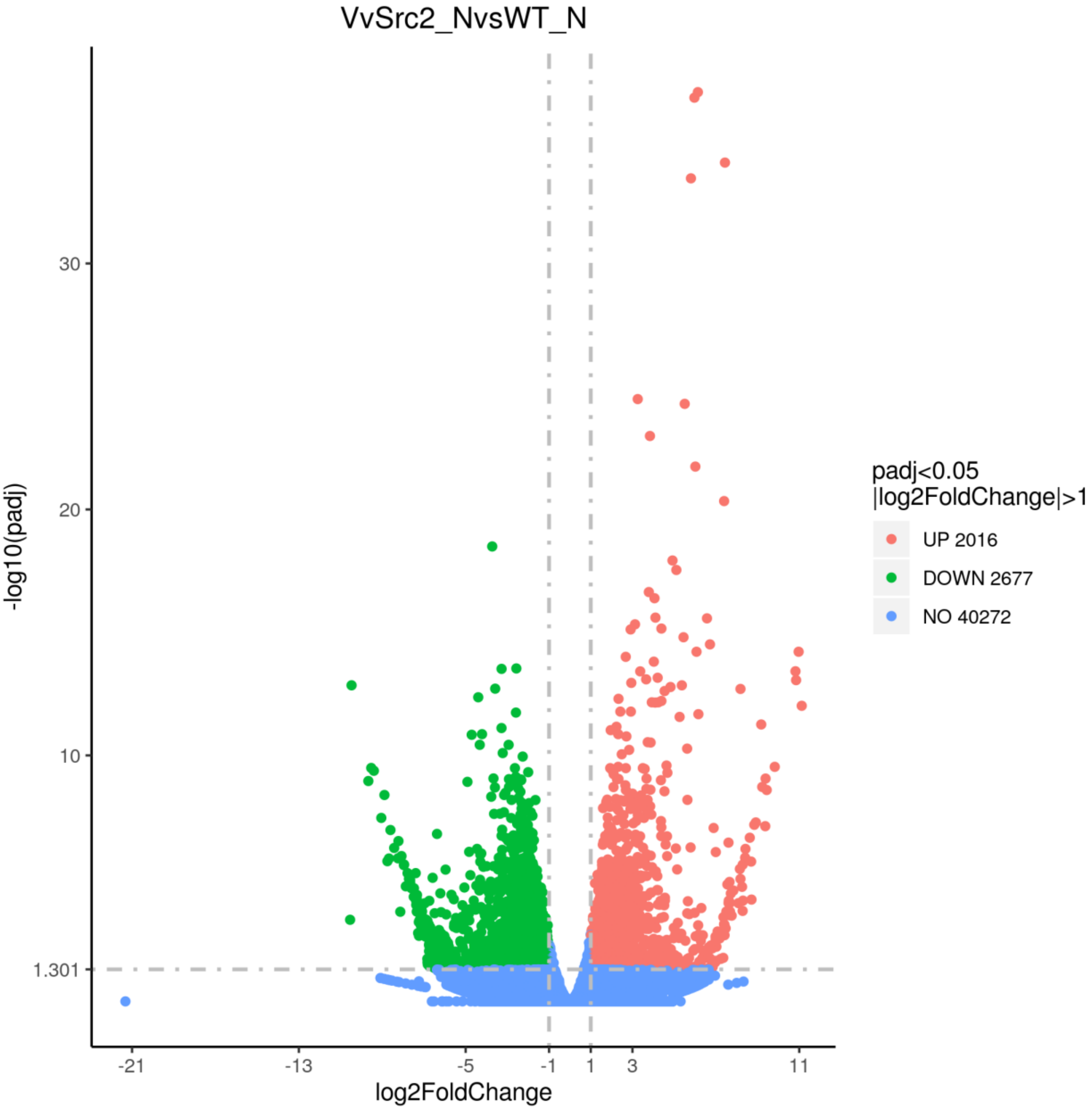

Supplementary Figure S6. Statistical analyses of differentially expressed genes (DEGs) between the wild type and the *VvSrc2*-transgenic line infected by *L. theobromae*.

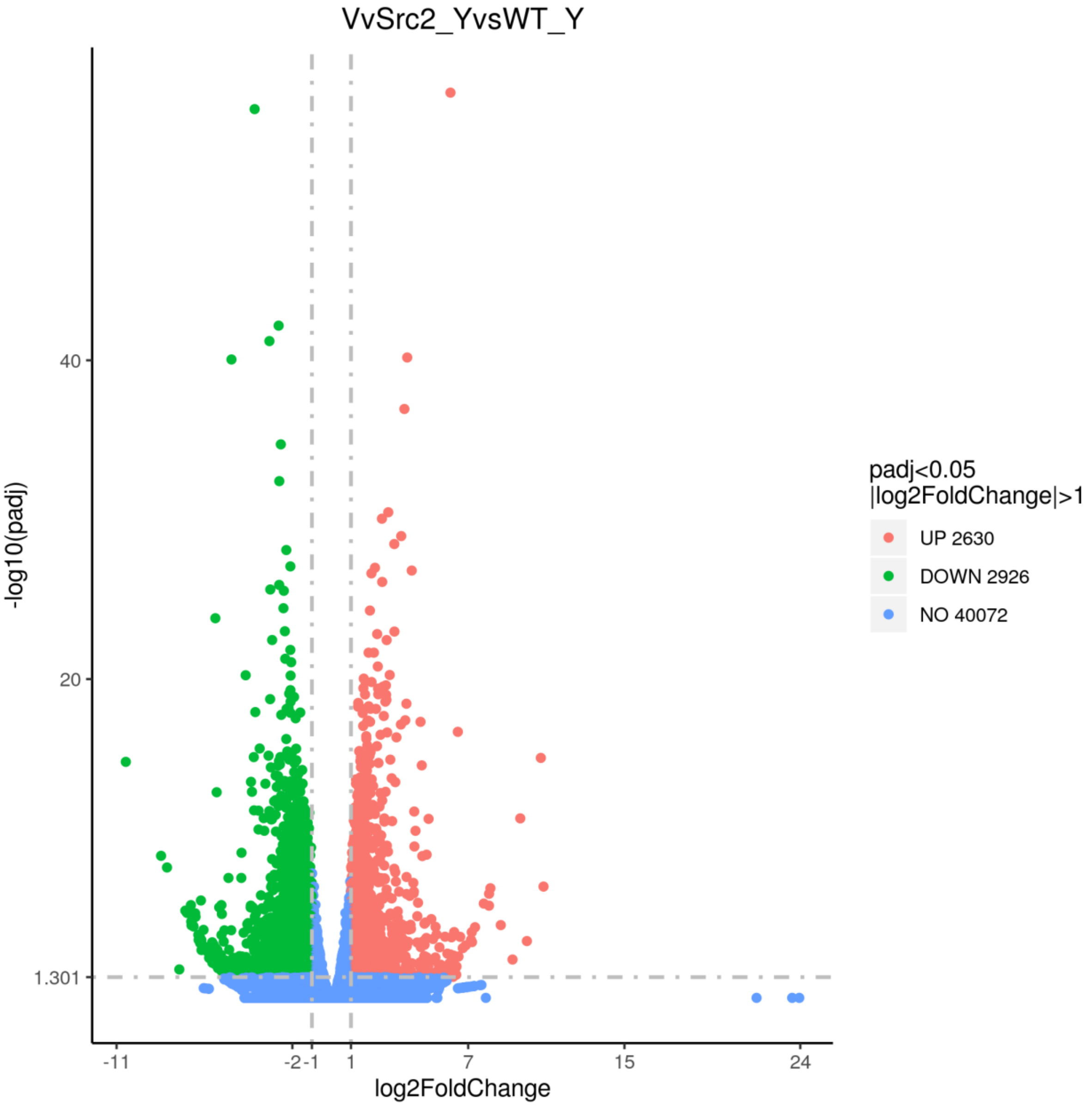

Supplementary Table S1. Systematic identification of LysM proteins in *L. theobromae*.

Protein name	Number of LysM domain	Signal peptide	Predicted to be an effector
LtLysM1	One	Yes	Yes
LtLysM2	One	Yes	Yes
LtLysM3	Three	Yes	Yes
LtLysM4	Four	Yes	No
LtLysM5	Three	Yes	No
LtLysM6	Four	No	No

Supplementary Table S2. The potential interacting targets of LtLysM2 obtained through cDNA library screening.

Protein name	Predicted Function Annotation	Subcellular Localization Prediction (Psort II)
VvChi4	Vitis vinifera class IV chitinase	Extracellular
VvTHF1	Vitis vinifera protein Thylakoid Formation 1	Chloroplastic
VvSrc2	Vitis vinifera protein SRC2-like	Cytoplasmic; Vesicles of secretory system
VvHDL1	Vitis vinifera probable 3-hydroxyisobutyrate dehydrogenase-like 1	Cytoplasmic; Nuclear
VvDSK2b	Vitis vinifera ubiquitin domain-containing protein DSK2b	Cytoplasmic; Nuclear
VvTBL10	Vitis vinifera protein trichome birefringence-like 10	Mitochondrial; Cytoplasmic

Supplementary Table S4. The potential interacting targets of VvSrc2 obtained through cDNA library screening.

Protein name	Predicted Function Annotation
VvGst1	*Vitis vinifera* glutathione S-transferase
VvUbp1	*Vitis vinifera* UBP1-associated protein 2C
VvBiP5	*Vitis vinifera* luminal-binding protein 5
VvMompp	*Vitis vinifera* mitochondrial outer membrane protein porin

Supplementary Table S5. Primers used in this study.

Primer name	Sequence （5'-3'）	Use of primer
LtLysM2SP-f	CGGAATTCATGACCAAGTTCACCACC	Used for the construction of *pSUC2: LtLysM2* vector
LtLysM2SP-r	TATCTCGAGGGGGAGGGCGGCGG	
LtLysM2OE-f	TTAAGCTTATGACCAAGTTCACCAC	Used for the construction of *LtLysM 2* overexpression vector
LtLysM2OE-r	CGGAATTCTTATGCCTCAACGCAG	
LtLysM2RNAi-sf	TATAAGCTTCAGGACGGCAACAACTG	Used for the construction of *LtLysM 2 silencing* vector
LtLysM2RNAi-sr	CGGAATTCCGTGTACGTGTACCAC	
LtLysM2RNAi-asf	CGGAATTCTGTAGAACTGGGTGACGG	
LtLysM2RNAi-asr	AAGGATCCCAGGACGGCAACAACTGC	
VvChi4$^{16-264}$AD-f	GCCATGGAGGCCAGTGAATTCATGGG AGCTGCAGTGGCTCAG	Used for the construction of *pGADT 7:VvChi4$^{16-264}$*vector
VvChi4$^{16-264}$AD-r	ATGCCCACCCGGGTGGAATTCTCAGCA AGTGAGGTTGTCAC	
VvChi4$^{16-55}$AD-f	GCCATGGAGGCCAGTGAATTCATGGG AGCTGCAGTGGCTCAG	Used for the construction of *pGADT 7:LtLysM2$^{16-55}$* vector
VvChi4$^{16-55}$AD-r	ATGCCCACCCGGGTGGAATTCTCATGA ATCACAAGGACCCGA	
VvChi4$^{55-216}$AD-f	GCCATGGAGGCCAGTGAATTCATGAGT AGCGGCAGTGGTAGC	Used for the construction of *pGADT 7:LtLysM2$^{55-216}$* vector

VvChi4$^{55-216}$AD-r	ATGCCCACCCGGGTGGAATTCTCAGCC TATGACAGAGTGAAC	
VvChi4$^{213-264}$AD-f	GCCATGGAGGCCAGTGAATTCATGGTC ATAGGCCAAGGTTT	Used for the construction of *pGADT 7:LtLysM2*$^{213-264}$ vector
VvChi4$^{213-264}$AD-r	ATGCCCACCCGGGTGGAATTCTCAGCA AGTGAGGTTGTCACCA	
VvSrc2$^{1-291}$AD-f	GCCATGGAGGCCAGTGAATTCATGGA GTACAGGACATTGG	Used for the construction of *pGADT 7:VvSrc2*$^{1-291}$ vector
VvSrc2$^{1-291}$AD-r	ATGCCCACCCGGGTGGAATTCTCAAAA GTCACCAAATCCAC	
VvSrc2$^{1-112}$AD-f	GCCATGGAGGCCAGTGAATTCATGGA GTACAGGACATTGG	Used for the construction of *pGADT 7:VvSrc2*$^{1-112}$ vector
VvSrc2$^{1-112}$AD-r	ATGCCCACCCGGGTGGAATTCTCAGAA CTGAATGGGTTTGGAT	
VvSrc2$^{113-291}$AD-f	GCCATGGAGGCCAGTGAATTCATGGTC AGTTATCAGGTCAGAA	Used for the construction of *pGADT 7:VvSrc2*$^{113-291}$ vector
VvSrc2$^{113-291}$AD-r	ATGCCCACCCGGGTGGAATTCTCAAAA GTCACCAAATCCAC	
VvSrc2$^{113-248}$AD-f	GCCATGGAGGCCAGTGAATTCATGGTC AGTTATCAGGTCAGAA	Used for the construction of *pGADT 7:VvSrc2*$^{113-248}$ vector
VvSrc2$^{113-248}$AD-r	ATGCCCACCCGGGTGGAATTCTCACTT GCTCTTCTTTGCCGGCT	

VvSrc2$^{272\text{-}291}$AD-f	GCCATGGAGGCCAGTGAATTCATGTCC GATGCAGCTGGTGG	Used for the construction of *pGADT 7:VvSrc2*$^{272\text{-}291}$ vector
VvSrc2$^{272\text{-}291}$AD-r	ATGCCCACCCGGGTGGAATTCTCAAAA GTCACCAAATCCAC	
LtLysM2$^{\Delta SP}$BD-f	CGGAATTCATGTTCCCTGCCTACT	Used for the construction of *pGBKT 7:LtLysM2*$^{\Delta SP}$ vector
LtLysM2$^{\Delta SP}$BD-r	CGGGATCCTTATGCCTCAACGCAG	
LtLysM2GST-f	CCGCGTGGATCCCCGGAATTCATGACC AAGTTCACCACC	Used for the construction of *pGEX-4T-1:LtLysM2* vector
LtLysM2GST-r	CTCGAGTCGACCCGGGAATTCTTATGC CTCAACGCAGC	
VvChi4MBP-f	GAGGGAAGGATTTCAGAATTCATGGC AGCCAAGCTACTA	Used for the construction of *pMAl-C4X:VvChi4* vector
VvChi4MBP-r	GACTCTAGAGGATCCGAATTCTCAGCA AGTGAGGTTGTC	
VvSrc2MBP-f	GAGGGAAGGATTTCAGAATTCATGGA GTACAGGACATTG	Used for the construction of *pMAl-C4X:VvSrc2* vector
VvSrc2MBP-r	GACTCTAGAGGATCCGAATTCTCAAAA GTCACCAAATCC	
VvUbp1-GST-f	CCGCGTGGATCCCCGGAATTCATGAAT TCTCAAACCCAAGTT	Used for the construction of *pGEX-4T-1: VvUbp1* vector

VvUbp1-GST-r	CTCGAGTCGACCCGGGAATTCTCATTG TTTGGCAAAGTAGTAAGG	
VvActinqRT-f	AACCCAAAGGCTAATCGTGAAA	Used for the internal control of qRT-PCR detection (grapevine)
VvActinqRT-r	TCCAGAGTCCAGAACAATACCA	
VvChi4qRT-f	GGTGCCTCTCATAACTACTG	Used for the transcript level detecti on of *VvChi4* gene
VvChi4qRT-r	CCATAATGCGGTCTTGAATG	
VvSrc2qRT-f	CCTCACTCTGTCCTTCAAG	Used for the transcript level detecti on of *VvSrc2* gene
VvSrc2qRT-r	GACCTGATAACTGACGAACT	
LtActinqRT-f	CCAAGTCCAACCGTGAGAA	Used for the internal control of qRT-PCR detection (*L. theobromae*)
LtActinqRT-r	GAAGCGTACAGCGACAGAA	
LtLysM2qRT-f	ATGACCAAGTTCACCACCC	Used for the transcript level detecti on of *LtLysM2* gene
LtLysM2qRT-r	AGAGAGTAGGCAGGGAAGG	
VvSrc2-GFP-f	GAGCTCGGTACCCGGGGATCCATGGA GTACAGGACATTGGA	Used for the transient expression of VvSrc2-GFP fusion protein
VvSrc2-GFP-r	GGTGTCGACTCTAGAGGATCCAAAGT CACCAAATCCACCGTCAT	
VvSrc2-mCherry-f	ACGGGGGACGAGCTCGGTACCATGGA GTACAGGACATTGGA	Used for the transient expression of VvSrc2-mCherry fusion protein
VvSrc2-mCherry-r	CACCATGGATCCCCGGGTACCAAAGTC ACCAAATCCACCGTCAT	

LtLLysM2-Cyfp-f	TCGAGCTCAAGCTTCGAATTCATGACC AAGTTCACCACC	Used for the transient expression of LtLLysM2-cYFP fusion protein
LtLLysM2-Cyfp-r	GTACCGTCGACTGCAGAATTCTGCCTC AACGCAGCTCCA	
Cyfp-LtLLysM2-f	GGTACCGCGGGCCCGGGATCCATGACC AAGTTCACCACC	Used for the transient expression of cYFP-LtLLysM2 fusion protein
Cyfp-LtLLysM2-r	GACTCTAGATCAGGTGGATCCTTATGC CTCAACGCAGCTCCA	
nYFP-VvSrc2-f	CGGTACCGCGGGCCCGGGATCCATGG AGTACAGGACATTGG	Used for the transient expression of nYFP-VvSrc2 fusion protein
nYFP-VvSrc2-r	GACTCTAGATCAGGTGGATCCTCAAAA GTCACCAAATCCACC	
VvSrc2-nYFP-f	TCGAGCTCAAGCTTCGAATTCATGGAG TACAGGACATTGG	Used for the transient expression of VvSrc2-nYFP fusion protein
VvSrc2-nYFP-r	GTACCGTCGACTGCAGAATTCAAAGTC ACCAAATCCACC	
VvUbp1-cyfp-f	TCGAGCTCAAGCTTCGAATTCATGAAT TCTCAAACCCAAGTT	Used for the transient expression of VvUbp1-cYFP fusion protein
VvUbp1-cyfp-r	GTACCGTCGACTGCAGAATTCTTGTTTG GCAAAGTAGTAAG	
cyfp-VvUbp1-f	GGTACCGCGGGCCCGGGATCCATGAA TTCTCAAACCCAAGTT	Used for the transient expression of cYFP-VvUbp1 fusion protein
cyfp-VvUbp1-r	GACTCTAGATCAGGTGGATCCTCATTG	

	TTTGGCAAAGTAGTAAG	
LOX2-qRT-f	AAGAGGAGTGGCTGTTGAAGA	Used for the transcript level detection of *LOX2* gene
LOX2-qRT-r	AGGCTTGGAGTTCTGTGTCT	
PR1-qRT-f	GCTGAGGGAAGTGGCGATTT	Used for the transcript level detection of *PR1* gene
PR1-qRT-R	TCCAACACGAACCGAGTTACG	
NPR3-qRT-f	GACATCAGCGGAAGCAGTAGT	Used for the transcript level detection of *NPR3* gene
NPR3-qRT-r	CTTAGCGTCGGCGAAGTAGT	
RbohD-qRT-f	CACCACCATCACCATCATTC	Used for the transcript level detection of *RbohD* gene
RbohD-qRT-r	ACGCATCATCATTGGACTTG	
RbohF-qRT-f	GTTGAGGGTGTGACGGGAAT	Used for the transcript level detection of *RbohF* gene
RbohF-qRT-r	TGACAAGAAGGTGGTGCGAAT	
NbActin-qRT-f	TGTTGGACTCTGGTGATGGT	Used for the internal control of qRT-PCR detection (*N. benthamiana*)
NbActin-qRT-r	ACGCTCGGTAAGGATCTTCAT	
