# Supplementary material for: Grapevine protein Src2 mediates plant disease resistance during Lasiodiplodia theobromae infection
Source: Plant Physiol. 2025 Nov 24;199(4):kiaf608. doi: 10.1093/plphys/kiaf608 (PMC12684713; doi:10.1093/plphys/kiaf608)
Supplement: kiaf608_Supplementary_Data [file kiaf608_supplementary_data.zip › Supplementary Table 1, 2, 4.pdf]

Table S1 Systematic identification of LysM proteins in *L. theobromae*.

Protein name	Number of LysM domain	Signal peptide	Predicted to be an effector
LtLysM1	One	Yes	Yes
LtLysM2	One	Yes	Yes
LtLysM3	Three	Yes	Yes
LtLysM4	Four	Yes	No
LtLysM5	Three	Yes	No
LtLysM6	Four	No	No

Table S2 The potential interacting targets of LtLysM2 obtained by cDNA library screening.

Protein name	Predicted Function Annotation	Subcellular Localization Prediction (Psort II)
VvChi4	Vitis vinifera class IV chitinase	Extracellular
VvTHF1	Vitis vinifera protein Thylakoid Formation 1	Chloroplastic
VvSrc2	Vitis vinifera protein SRC2-like	Cytoplasmic; Vesicles of secretory system
VvHDL1	Vitis vinifera probable 3-hydroxyisobutyrate dehydrogenase-like 1	Cytoplasmic; Nuclear
VvDSK2b	Vitis vinifera ubiquitin domain-containing protein DSK2b	Cytoplasmic; Nuclear
VvTBL10	Vitis vinifera protein trichome birefringence-like 10	Mitochondrial; Cytoplasmic

Table S4 The potential interacting targets of VvSrc2 obtained by cDNA library screening.

Protein name	Predicted Function Annotation
VvGst1	*Vitis vinifera* glutathione S-transferase
VvUbp1	*Vitis vinifera* UBP1-associated protein 2C
VvBiP5	*Vitis vinifera* luminal-binding protein 5
VvMompp	*Vitis vinifera* mitochondrial outer membrane protein porin
