# Supplementary material for: Grapevine protein Src2 mediates plant disease resistance during Lasiodiplodia theobromae infection
Source: Plant Physiol. 2025 Nov 24;199(4):kiaf608. doi: 10.1093/plphys/kiaf608 (PMC12684713; doi:10.1093/plphys/kiaf608)
Supplement: kiaf608_Supplementary_Data [file kiaf608_supplementary_data.zip › Supplementary Table 5.pdf]

Table S5 Primers used in this study.

Primer name	Sequence （5’-3’）	Use of primer
LtLysM2SP-f	CGGAATTCATGACCAAGTTCACCACC	Used for the construction of *pSUC2: LtLysM2* vector
LtLysM2SP-r	TATCTCGAGGGGGAGGGCGGCGG	
LtLysM2OE-f	TTAAGCTTATGACCAAGTTCACCAC	Used for the construction of *LtLysM 2* overexpression vector
LtLysM2OE-r	CGGAATTCTTATGCCTCAACGCAG	
LtLysM2RNAi-sf	TATAAGCTTCAGGACGGCAACAACTG	Used for the construction of *LtLysM 2 silencing* vector
LtLysM2RNAi-sr	CGGAATTCCGTGTACGTGTACCAC	
LtLysM2RNAi-asf	CGGAATTCTGTAGAACTGGGTGACGG	
LtLysM2RNAi-asr	AAGGATCCCAGGACGGCAACAACTGC	
VvChi4$^{16\text{-}264}$AD-f	GCCATGGAGGCCAGTGAATTCATGGG AGCTGCAGTGGCTCAG	Used for the construction of *pGADT 7:VvChi4$^{16\text{-}264}$*vector
VvChi4$^{16\text{-}264}$AD-r	ATGCCCACCCGGGTGGAATTCTCAGCA AGTGAGGTTGTCAC	
VvChi4$^{16\text{-}55}$AD-f	GCCATGGAGGCCAGTGAATTCATGGG AGCTGCAGTGGCTCAG	Used for the construction of *pGADT 7:LtLysM2$^{16\text{-}55}$* vector
VvChi4$^{16\text{-}55}$AD-r	ATGCCCACCCGGGTGGAATTCTCATGA ATCACAAGGACCCGA	
VvChi4$^{55\text{-}216}$AD-f	GCCATGGAGGCCAGTGAATTCATGAGT AGCGGCAGTGGTAGC	Used for the construction of *pGADT 7:LtLysM2$^{55\text{-}216}$* vector

VvChi4$^{55-216}$AD-r	ATGCCCACCCGGGTGGAATTCTCAGCC TATGACAGAGTGAAC	
VvChi4$^{213-264}$AD-f	GCCATGGAGGCCAGTGAATTCATGGTC ATAGGCCAAGGTTT	Used for the construction of *pGADT 7:LtLysM2*$^{213-264}$ vector
VvChi4$^{213-264}$AD-r	ATGCCCACCCGGGTGGAATTCTCAGCA AGTGAGGTTGTCACCA	
VvSrc2$^{1-291}$AD-f	GCCATGGAGGCCAGTGAATTCATGGA GTACAGGACATTGG	Used for the construction of *pGADT 7:VvSrc2*$^{1-291}$ vector
VvSrc2$^{1-291}$AD-r	ATGCCCACCCGGGTGGAATTCTCAAAA GTCACCAAATCCAC	
VvSrc2$^{1-112}$AD-f	GCCATGGAGGCCAGTGAATTCATGGA GTACAGGACATTGG	Used for the construction of *pGADT 7:VvSrc2*$^{1-112}$ vector
VvSrc2$^{1-112}$AD-r	ATGCCCACCCGGGTGGAATTCTCAGAA CTGAATGGGTTTGGAT	
VvSrc2$^{113-291}$AD-f	GCCATGGAGGCCAGTGAATTCATGGTC AGTTATCAGGTCAGAA	Used for the construction of *pGADT 7:VvSrc2*$^{113-291}$ vector
VvSrc2$^{113-291}$AD-r	ATGCCCACCCGGGTGGAATTCTCAAAA GTCACCAAATCCAC	
VvSrc2$^{113-248}$AD-f	GCCATGGAGGCCAGTGAATTCATGGTC AGTTATCAGGTCAGAA	Used for the construction of *pGADT 7:VvSrc2*$^{113-248}$ vector
VvSrc2$^{113-248}$AD-r	ATGCCCACCCGGGTGGAATTCTCACTT GCTCTTCTTTGCCGGCT	

VvSrc2$^{272-291}$AD-f	GCCATGGAGGCCAGTGAATTCATGTCCGATGCAGCTGGTGG	Used for the construction of *pGADT7:VvSrc2$^{272-291}$* vector
VvSrc2$^{272-291}$AD-r	ATGCCCACCCGGGTGGAATTCTCAAAAGTCACCAAATCCAC	
LtLysM2$^{\Delta SP}$BD-f	CGGAATTCATGTTCCCTGCCTACT	Used for the construction of *pGBKT7:LtLysM2$^{\Delta SP}$* vector
LtLysM2$^{\Delta SP}$BD-r	CGGGATCCTTATGCCTCAACGCAG	
LtLysM2GST-f	CCGCGTGGATCCCCGGAATTCATGACCAAGTTCACCACC	Used for the construction of *pGEX-4T-1:LtLysM2* vector
LtLysM2GST-r	CTCGAGTCGACCCGGGAATTCTTATGCCTCAACGCAGC	
VvChi4MBP-f	GAGGGAAGGATTTCAGAATTCATGGCAGCCAAGCTACTA	Used for the construction of *pMAl-C4X:VvChi4* vector
VvChi4MBP-r	GACTCTAGAGGATCCGAATTCTCAGCAAGTGAGGTTGTC	
VvSrc2MBP-f	GAGGGAAGGATTTCAGAATTCATGGAGTACAGGACATTG	Used for the construction of *pMAl-C4X:VvSrc2* vector
VvSrc2MBP-r	GACTCTAGAGGATCCGAATTCTCAAAAGTCACCAAATCC	
VvUbp1-GST-f	CCGCGTGGATCCCCGGAATTCATGAATTCTCAAACCCAAGTT	Used for the construction of *pGEX-4T-1: VvUbp1* vector

VvUbp1-GST-r	CTCGAGTCGACCCGGGAATTCTCATTG TTTGGCAAAGTAGTAAGG	
VvActinqRT-f	AACCCAAAGGCTAATCGTGAAA	Used for the internal control of qRT-PCR detection (grapevine)
VvActinqRT-r	TCCAGAGTCCAGAACAATACCA	
VvChi4qRT-f	GGTGCCTCTCATAACTACTG	Used for the transcript level detecti on of *VvChi4* gene
VvChi4qRT-r	CCATAATGCGGTCTTGAATG	
VvSrc2qRT-f	CCTCACTCTGTCCTTCAAG	Used for the transcript level detecti on of *VvSrc2* gene
VvSrc2qRT-r	GACCTGATAACTGACGAACT	
LtActinqRT-f	CCAAGTCCAACCGTGAGAA	Used for the internal control of qRT-PCR detection (*L. theobromae*)
LtActinqRT-r	GAAGCGTACAGCGACAGAA	
LtLysM2qRT-f	ATGACCAAGTTCACCACCC	Used for the transcript level detecti on of *LtLysM2* gene
LtLysM2qRT-r	AGAGAGTAGGCAGGGAAGG	
VvSrc2-GFP-f	GAGCTCGGTACCCGGGGATCCATGGA GTACAGGACATTGGA	Used for the transient expression of VvSrc2-GFP fusion protein
VvSrc2-GFP-r	GGTGTCGACTCTAGAGGATCCAAAGT CACCAAATCCACCGTCAT	
VvSrc2-mCherry-f	ACGGGGGACGAGCTCGGTACCATGGA GTACAGGACATTGGA	Used for the transient expression of VvSrc2-mCherry fusion protein
VvSrc2-mCherry-r	CACCATGGATCCCCGGGTACCAAAGTC ACCAAATCCACCGTCAT	

LtLLysM2-Cyfp-f	TCGAGCTCAAGCTTCGAATTCATGACC AAGTTCACCACC	Used for the transient expression of LtLLysM2-cYFP fusion protein
LtLLysM2-Cyfp-r	GTACCGTCGACTGCAGAATTCTGCCTC AACGCAGCTCCA	
Cyfp-LtLLysM2-f	GGTACCGCGGGCCCGGGATCCATGACC AAGTTCACCACC	Used for the transient expression of cYFP-LtLLysM2 fusion protein
Cyfp-LtLLysM2-r	GACTCTAGATCAGGTGGATCCTTATGC CTCAACGCAGCTCCA	
nYFP-VvSrc2-f	CGGTACCGCGGGCCCGGGATCCATGG AGTACAGGACATTGG	Used for the transient expression of nYFP-VvSrc2 fusion protein
nYFP-VvSrc2-r	GACTCTAGATCAGGTGGATCCTCAAAA GTCACCAAATCCACC	
VvSrc2-nYFP-f	TCGAGCTCAAGCTTCGAATTCATGGAG TACAGGACATTGG	Used for the transient expression of VvSrc2-nYFP fusion protein
VvSrc2-nYFP-r	GTACCGTCGACTGCAGAATTCAAAGTC ACCAAATCCACC	
VvUbp1-cyfp-f	TCGAGCTCAAGCTTCGAATTCATGAAT TCTCAAACCCAAGTT	Used for the transient expression of VvUbp1-cYFP fusion protein
VvUbp1-cyfp-r	GTACCGTCGACTGCAGAATTCTTGTTTG GCAAAGTAGTAAG	
cyfp-VvUbp1-f	GGTACCGCGGGCCCGGGATCCATGAA TTCTCAAACCCAAGTT	Used for the transient expression of cYFP-VvUbp1 fusion protein
cyfp-VvUbp1-r	GACTCTAGATCAGGTGGATCCTCATTG	

	TTTGGCAAAGTAGTAAG	
LOX2-qRT-f	AAGAGGAGTGGCTGTTGAAGA	Used for the transcript level detection of *LOX2* gene
LOX2-qRT-r	AGGCTTGGAGTTCTGTGTCT	
PR1-qRT-f	GCTGAGGGAAGTGGCGATTT	Used for the transcript level detection of *PR1* gene
PR1-qRT-R	TCCAACACGAACCGAGTTACG	
NPR3-qRT-f	GACATCAGCGGAAGCAGTAGT	Used for the transcript level detection of *NPR3* gene
NPR3-qRT-r	CTTAGCGTCGGCGAAGTAGT	
RbohD-qRT-f	CACCACCATCACCATCATTC	Used for the transcript level detection of *RbohD* gene
RbohD-qRT-r	ACGCATCATCATTGGACTTG	
RbohF-qRT-f	GTTGAGGGTGTGACGGGAAT	Used for the transcript level detection of *RbohF* gene
RbohF-qRT-r	TGACAAGAAGGTGGTGCGAAT	
NbActin-qRT-f	TGTTGGACTCTGGTGATGGT	Used for the internal control of qRT-PCR detection (*N. benthamiana*)
NbActin-qRT-r	ACGCTCGGTAAGGATCTTCAT	
